# Enhanced Media Optimize Bovine Myogenesis in 2D and 3D Models for Cultivated Meat Applications

**DOI:** 10.1002/advs.202413998

**Published:** 2025-07-28

**Authors:** Christine L. Trautmann, Adhideb Ghosh, Ali Kerem Kalkan, Falko Noé, Ori Bar‐Nur

**Affiliations:** ^1^ Laboratory of Regenerative and Muscle Biology, Institute of Human Movement Sciences and Sport Department of Health Sciences and Technology ETH Zurich Schwerzenbach 8603 Switzerland; ^2^ Functional Genomics Center Zurich ETH Zurich and University of Zurich Zurich 8057 Switzerland

**Keywords:** 3D tissue‐engineered muscles, bovine myogenesis, cultivated meat, multiomics, single‐cell omics, skeletal muscle differentiation, small molecules

## Abstract

Livestock farming and conventional meat production pose significant environmental, health, and animal welfare challenges. In seeking sustainable alternative solutions, cultivated meat technology typically utilizes differentiation of myogenic progenitor cells (MPCs) into muscle cells for in vitro meat production. However, understanding the molecular determinants governing MPC differentiation into muscle cells, and the potential enhancement of this process through modulation of signaling pathways, remains limited. Herein, we characterized the molecular landscape associated with bovine MPC differentiation in vitro by employing multiomics, and explored its augmentation by small molecules, together leading to identification of media that enhanced myogenic differentiation compared with conventional methods in both 2D cultures and tissue‐engineered 3D skeletal muscle constructs. Through bulk and single‐cell transcriptomics and proteomics, we compared conventional and enhanced differentiation media, demonstrating that the enhanced media gave rise to unique progenitor‐like cell populations, while simultaneously promoting differentiation into myocytes and contractile myotubes expressing a wide array of myogenic markers that more closely resemble bovine muscle cells in vivo. The improved method for promoting myogenic differentiation in 2D and 3D formats, together with the corresponding molecular roadmap, may prove valuable for cultivated meat applications.

## Graphical abstract

This study uses multiomics approaches to characterize bovine myogenesis in vitro, identifying media components that enhance myogenic progenitor cell differentiation in 2D and 3D models. Notably, the enhanced conditions promoted formation of unique cell populations and contractile muscle cells expressing mature myogenic markers. The optimized method, along with the corresponding molecular roadmap, provides a valuable resource for advancing cultivated meat.

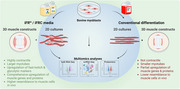

## 1 Introduction

Human diets have evolved to contain both animal‐ and non‐animal‐based products. While humans can live on plant‐based diets, many societies are still heavily dependent on the consumption of animal‐based products, such as meat or dairy. This dependency is predominantly a result of the nutrient‐rich content of meat, as well as cultural and flavor‐related preferences.^[^ ^1^, ^2^ ^]^ However, animal‐based diets impose unfavorable constraints on the environment, climate, and animal welfare, primarily due to land requirements, significant contribution to greenhouse gas emissions, and involvement in the unethical use or slaughter of animals.^[^ ^1^, ^3^, ^4^ ^]^ In particular, greenhouse gas emissions, a byproduct of animal use in the meat industry, are considered a major contributor to the rising world temperatures, leading to deleterious effects on the environment and human health.^[^ ^1^, ^3^, ^4^ ^]^

Meat is primarily composed of skeletal muscle, a tissue that constitutes ≈30%–40% of normal human body mass. This tissue is mostly comprised of multinucleated muscle fibers (or myofibers) that contract to generate locomotion.^[^ ^5^ ^]^ In addition, this tissue contains muscle stem cells known as “satellite cells”, which are quiescent under homeostatic conditions, and activate to repair skeletal muscle tissue upon damage.^[^ ^6^, ^7^, ^8^ ^]^ This process occurs through satellite cell activation into proliferating myoblasts that differentiate into fusion‐competent myocytes that merge with muscle fibers for tissue repair.^[^ ^9^, ^10^ ^]^ The myogenic differentiation program that results in muscle regeneration is characterized by specific expression of transcription factors including *Pax7* and *Myf5* in satellite cells, *MyoD* in myoblasts, and *Myog* and myosin heavy chain (*Myh*) isoforms in myocytes and multinucleated muscle cells.^[^ ^5^, ^9^, ^10^ ^]^ Additionally, muscle tissue contains other cell types, including immune and endothelial cells, motor neurons, as well as fibroblasts and fibro‐adipogenic progenitors (FAPs) which play a role in regeneration.^[^ ^11^ ^]^

The in vitro generation of meat‐like products, commonly referred to as “cultivated meat”, “cultured meat” or “cell‐based meat”, is a practice by which MPCs, typically myoblasts, are expanded to high numbers in vitro and induced to differentiate into multinucleated fibers (often referred to as “myotubes”), which can serve as a source of cultivated meat products.^[^ ^4^, ^12^, ^13^, ^14^ ^]^ However, additional cell types, such as chicken fibroblasts, have also been reported as a potential source for cultivated meat.^[^ ^15^ ^]^ Furthermore, to obtain 3D tissue‐like structures that mimic the characteristics of a steak or filet, tissue engineering techniques are required together with the addition of other cell types such as adipocytes for flavor.^[^ ^16^, ^17^ ^]^ Notably, the initial phase of muscle cell line derivation is a critical step, as it aims to achieve efficient and cost‐effective cell proliferation to accumulate a biomass.^[^ ^12^, ^18^ ^]^ Historically, seminal works in rodents unveiled the in vitro culture conditions required for myoblast propagation in high serum together with basic fibroblast growth factor (bFGF), and their differentiation into myotubes via serum and bFGF withdrawal.^[^ ^19^, ^20^, ^21^ ^]^ These efforts yielded widely used rodent myoblast lines such as L6 and C2C12, which capture, to a certain degree, myogenesis in vitro.^[^ ^20^, ^21^, ^22^ ^]^ However, it has also been reported that primary myoblasts lose myogenic capacities following in vitro propagation in comparison to satellite cells.^[^ ^23^ ^]^

To address this limitation, a recently developed protocol reported the conversion of mouse fibroblasts into induced myogenic progenitor cells (iMPCs) by overexpression of *MyoD* in concert with three small molecules, the cyclic AMP agonist Forskolin (F), the TGF‐β receptor inhibitor RepSox (R) and the GSK3‐β inhibitor CHIR99021 (C) (abbreviated as FRC).^[^ ^24^ ^]^ Combined administration of FR or FRC, but not other combinations of F, R and C, enabled the propagation of proliferative iMPCs comprised of muscle stem cells along with differentiated progeny including contractile myotubes.^[^ ^24^ ^]^ Furthermore, this heterogeneous culture effectively captures myogenesis in vitro and uniquely contains Pax7‐expressing mouse muscle stem cells that resemble satellite cells in vivo.^[^ ^25^, ^26^, ^27^ ^]^ For cultivated meat purposes, adapting this approach to domesticated animals could be of interest considering the limitations of committed myoblasts in expanding and differentiating, which may require their immortalization.^[^ ^28^ ^]^ However, whether this treatment can induce myogenic differentiation or de‐differentiation in domesticated animal myoblasts has not been investigated. In this study, we set out to explore if iMPC medium, supplemented with FR or FRC, can enhance bovine myogenesis in vitro at both the progenitor and differentiated cell levels. Specifically, we sought to determine whether this treatment can give rise to progenitor cells, or differentiated myogenic cells that more closely resemble in vivo‐derived muscle cells compared with a conventional differentiation protocol, in both 2D and tissue‐engineered 3D models. Facilitating an improved method for bovine muscle cell differentiation in vitro may carry potential implications for cultivated meat generation.

## 2 Results

### 2.1 Characterizing Conventional Bovine Myoblast Differentiation In Vitro with Multiomics

**Figure 1:**
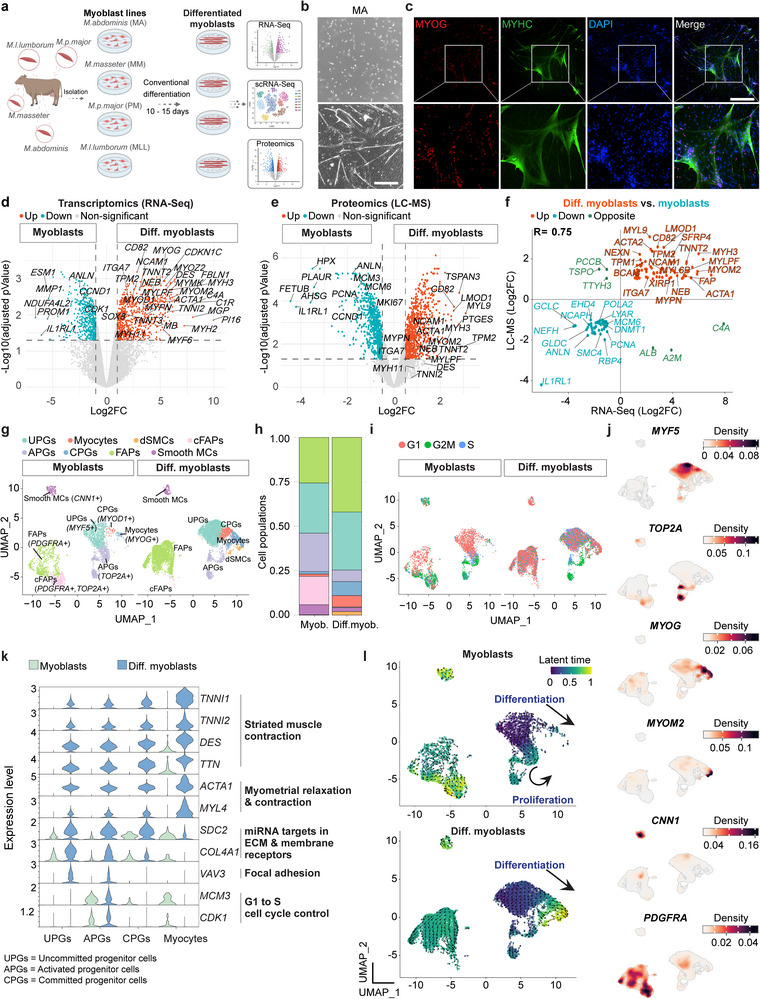
Dissecting conventional bovine myoblast differentiation utilizing multiomics. a) Schematic of experimental design. Four bovine muscle types were used as a source for derivation of myoblast lines in conventional myoblast medium containing high serum (30%) and bFGF. A conventional differentiation medium (Conv.Diff.) containing low serum (2%) and no bFGF was used to induce myoblast differentiation. The specified multiomics assays were used to characterize myoblasts and differentiated myoblasts. b) Representative phase contrast images of MA muscle‐derived bovine myoblasts a day after splitting (day 0, top) or in Conv.Diff. medium (day 10, bottom). Note formation of elongated myotubes at day 10. Look‐up tables (LUTs) were equally adjusted. Scale bar, 500 µm. c) Representative immunofluorescence images of MM muscle‐derived myoblasts subjected to the Conv.Diff. condition and stained for the indicated differentiation markers at day 10. Nuclei were stained with DAPI. Scale bar, 500 µm. d) Volcano plot based on bulk RNA‐Seq showing DEGs between the indicated conditions. Significant DEGs (|log2FC| > 1, FDR < 0.05) are shown as colored dots. N = 3 myoblast lines per group, derived from three different muscle types. The differentiated myoblasts were analyzed at day 15. e) Volcano plot based on LC‐MS showing DEPs between the indicated conditions. Significant DEPs (|log2FC| > 1, FDR < 0.05) are shown as colored dots. N = 2 for myoblasts and N = 4 for differentiated myoblasts. The differentiated myoblasts were analyzed at day 10. f) Scatter plot showing an integrative transcriptome/proteome correlation analysis between significant DEGs and DEPs that are upregulated in differentiated myoblasts versus myoblasts. A Pearson's correlation co‐efficient R for DEGs and DEPs is indicated. g) UMAP projection based on scRNA‐Seq of integrated cells from the indicated samples colored by specific cell types. An MA myoblast line was used for this analysis. The differentiated myoblasts were analyzed at day 10. h) Bar plots showing relative cell type distribution based on the UMAP projection shown in (g). i) UMAP projection of integrated cells colored by cell cycle states. j) UMAP projection of integrated cells colored by density expression gradient for the specified marker genes. An integration of “myoblasts” and “differentiated myoblasts” datasets was used for this analysis. k) Violin plots demonstrating the expression of DEGs associated with the indicated GO terms in the specified cell populations. l) UMAP projection of integrated cells colored by latent time as calculated by RNA velocity.

We commenced this study with the aim of delineating the molecular architecture of bovine myoblasts during differentiation into myocytes and multinucleated myotubes, employing a widely used method for myoblast propagation and differentiation.^[^ ^19^, ^29^ ^]^ For molecular characterization, we utilized multiomics assays including bulk RNA‐sequencing (RNA‐Seq), single‐cell RNA‐sequencing (scRNA‐Seq) and bulk proteomics through liquid chromatography‐mass spectrometry (LC‐MS). As a myogenic cell source, we derived multiple myoblast lines from four bovine muscles: *M. abdominis* (MA), *M. masseter* (MM), *M.p. major* (PM), and *M.l. lumborum* (MLL) (**Figure** **1**a,b; Figure S1a and Table S1, Supporting Information). For MA and MM, we derived three myoblast lines from three different cows, whereas for PM and MLL muscles we derived one myoblast line per cow (Table S1, Supporting Information). We generated myoblasts from various muscle types as we aimed to investigate the differentiation potential of myoblasts originating from different muscles (Figure 1a). Myoblast cultures were established using a commonly used pre‐plating technique and cultured in high serum (30%) supplemented with bFGF, a method which ensures extensive myoblast proliferation.^[^ ^19^, ^29^ ^]^ Of note, bovine myoblast lines generated via this approach also contain proliferative FAPs, fibroblasts and other cell types, thus representing a heterogeneous culture.^[^ ^30^, ^31^ ^]^ To differentiate the myoblasts into myotubes, we employed a widely used conventional differentiation medium (“Conv.Diff.”) containing low serum (2%) and no bFGF.^[^ ^19^, ^32^ ^]^ For this, myoblasts were plated for 4 days in conventional myoblast medium before replacement with the Conv.Diff. medium for 6 days. This effort readily gave rise to elongated myotubes that expressed the differentiation markers MYOG and MYHC (Figure 1b,c).

To molecularly characterize these myogenic cultures, we subjected myoblasts to the Conv.Diff. condition and performed transcriptomic and proteomic analyses at days 10–15 using RNA‐Seq and LC‐MS, comparing them to myoblasts that have been maintained in myoblast medium (Table S2, Supporting Information). We documented a suite of differentially expressed genes (DEGs) and proteins (DEPs) that were upregulated in differentiated myoblasts under the Conv.Diff. condition, including the skeletal muscle markers *MYOG*, *MYH3*, *MYMK*, *ACTA1*, *TPM2* and *NEB* (Figure 1d–f). Based on RNA‐Seq, the top upregulated DEGs were annotated with the gene ontology (GO) term “*Striated muscle contraction*” (Figure S1b, Supporting Information). Additionally, non‐differentiated myoblasts were enriched for various markers including *ESM1*, *GLDC*, *FETUB* and the proliferation and cell cycle regulators *PCNA*, *MKI67*, *CCND1*, *MCM6* and more, in accordance with their progenitor state (Figure 1d–f).

As the multiomics assays were performed on bulk myogenic cultures, we next employed scRNA‐Seq to characterize the mononucleated cell populations within myoblasts and differentiated myoblasts. We identified 8 cell populations through the analysis of 11,161 cells that were present in either MA‐derived myoblasts or differentiated myoblasts, including *MYF5* ^+^ uncommitted progenitors (“UPGs”), *MYF5* ^+^/*TOP2A* ^+^ activated progenitors (“APGs”), *MYOD1* ^+^/*MYOG* ^+^ committed progenitors (“CPGs”), *MYOG* ^+^/*MYLPF* ^+^ “myocytes”, *MYH3* ^+^/*MYOM2* ^+^ differentiated skeletal muscle cells (“dSMCs”), *PDGFRA* ^+^/*BGN* ^+^ “FAPs”, *PDGFRA* ^+^/*TOP2A* ^+^ cycling FAPs (“cFAPs”), and *CNN1* ^+^/*ACTG2* ^+^ smooth muscle‐like cells (“Smooth MCs”) (Figure 1g–j; Figure S1c–e, Supporting Information). Notably, only differentiated myoblasts contained dSMCs and more CPGs and myocytes, whereas myoblasts contained more APGs and cFAPs, in accordance with their proliferative cell state (Figure 1g–j; Figure S1c–e, Supporting Information).

Next, we investigated which genes were differentially expressed by scRNA‐Seq in the various cell populations, aiming to identify upregulated GO terms representing unique biological processes. The CPGs and myocyte populations were characterized by the GO terms “*Striated muscle contraction*” and “*Myometrial relaxation and contraction*”, while APGs were characterized by the GO term “*G1 to S cell cycle control*” (Figure 1k). Furthermore, the expression of established muscle genes and their respective GO terms gradually increased during the differentiation course (Figure 1k; Figure S1f, Supporting Information). Finally, to infer temporal gene expression dynamics in myoblasts and differentiated myoblasts, we performed an RNA velocity analysis based on scRNA‐Seq, a method which accounts for relative abundance of spliced and unspliced mRNA molecules.^[^ ^33^ ^]^ Through this approach, we confirmed the differentiation cascade observed in differentiated myoblast cultures, revealing a unidirectional trajectory toward dSMCs, whereas myoblasts showed enhanced transcriptional dynamics in proliferating cell populations such as cFAPs and APGs (Figure 1l). In summary, we utilized multiomics to dissect the transcriptome and proteome landscape of bovine myoblasts cultured under conventional myoblast medium, and differentiated using one of the most common protocols in the field, documenting genes, proteins and pathways unique to each cellular state.

### 2.2 Enhanced Myoblast Differentiation into Myotubes using Small Molecules

**Figure 2:**
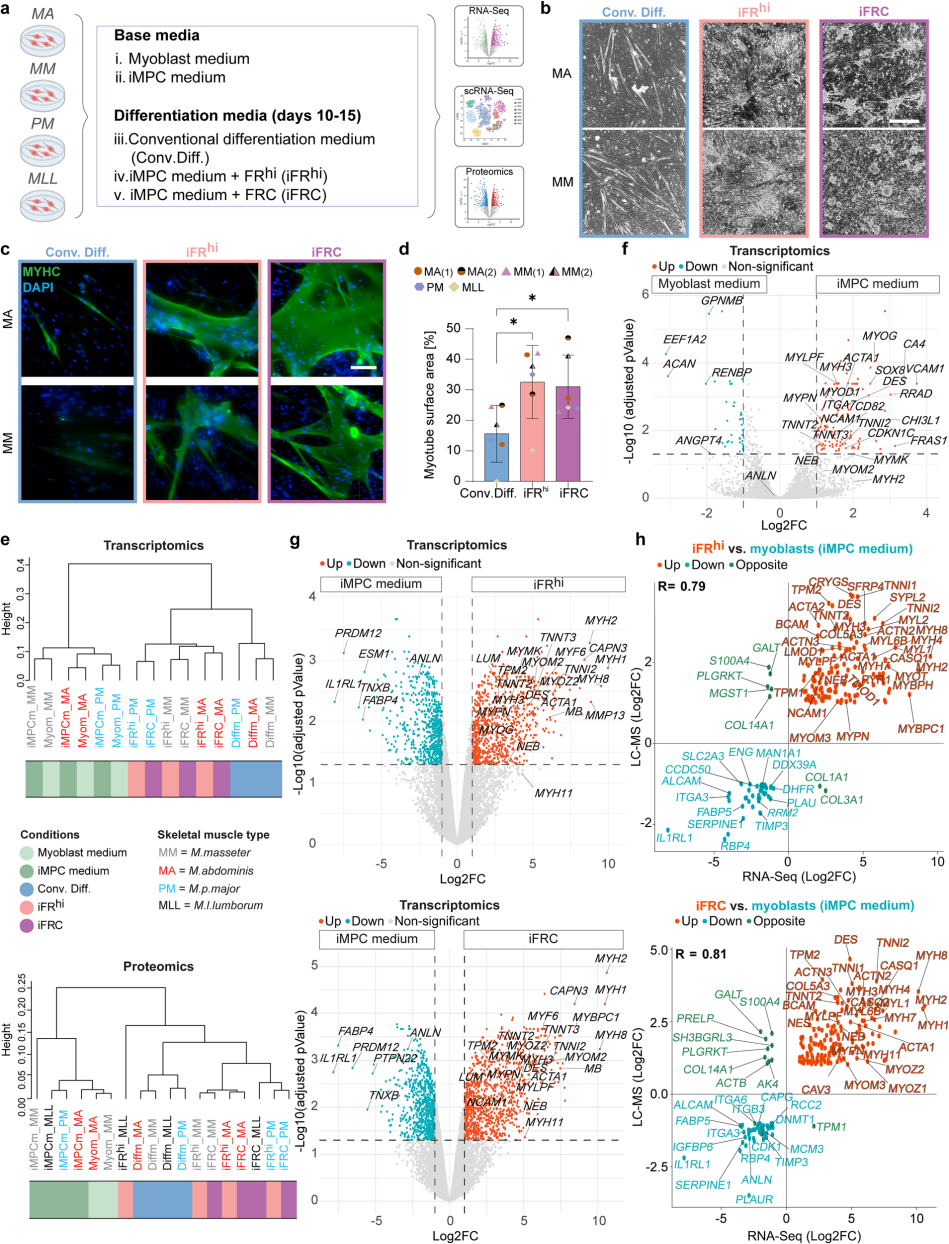
Small molecule administration enhances bovine myoblast differentiation. a) Schematic of the experimental layout to test the effects of media and small molecules on bovine myoblast differentiation. b) Representative phase contrast images of the specified myoblasts cultured under the indicated conditions at day 10. LUTs for MA and MM were individually adjusted. Scale bar, 500 µm. c) Representative immunofluorescence images for MYHC in differentiated myoblasts subjected to the indicated conditions at day 10. Nuclei were stained with DAPI. LUTs were individually adjusted. Scale bar, 100 µm. d) A graph showing quantification of myotube surface area (in percentage) in differentiated myoblasts subjected to the indicated conditions on day 10. Note an increase in the surface area of myotubes with small molecule treatment. N = 4 random images per condition using six myoblast lines derived from four different muscle types. Error bars denote standard deviation (SD). Statistical significance was determined by one‐way analysis of variance (ANOVA), followed by post‐hoc Dunnett's test, ^*^p ≤ 0.05. e) Dendrogram based on RNA‐Seq (top) and LC‐MS (bottom) showing the hierarchical clustering of the indicated cell lines and conditions. The cultures were analyzed at day 15 (RNA‐Seq) or day 10 (LC‐MS). Samples are colored by different conditions and annotated by skeletal muscle type. f) Volcano plot based on RNA‐Seq showing DEGs between the indicated conditions. Significant DEGs (|log2FC| > 1, p < 0.01) are shown as colored dots. N = 3 myoblast lines per group, derived from three different muscle types. g) Volcano plots based on RNA‐Seq showing DEGs between the indicated conditions. Note the upregulation of myogenic markers in the iFR^hi^ and iFRC conditions. Significant DEGs (|log2FC| > 1, FDR < 0.05) are shown as colored dots. N = 3 myoblast lines per group, derived from three different muscle types. The cultures were analyzed at day 15. h) Scatter plots demonstrating an integrative transcriptome/proteome correlation analysis between significant DEGs and DEPs upregulated in the iFR^hi^ and iFRC conditions versus myoblasts cultured in iMPC medium. A Pearson's correlation co‐efficient R for DEGs and DEPs is indicated. The cultures were analyzed at day 15 (RNA‐Seq) or day 10 (LC‐MS).

Following the molecular characterization of bovine myoblast differentiation under a conventional protocol, we next aimed to dissect the effect of cell medium supplements, as well as small molecule administration, on the differentiation capacities of myoblast lines derived from different muscle types (Table S2, Supporting Information). Specifically, we compared myoblasts cultured under conventional myoblast medium^[^ ^29^ ^]^ (Figure 1) to iMPC medium consisting of 10% serum, 10% serum replacement and bFGF, with and without FR or FRC supplementation (**Figure** **2**a).^[^ ^24^, ^34^ ^]^ We chose previously reported small molecule concentrations for iMPC medium containing FRC (“iFRC”) (5, 5 and 3 µM, respectively)^[^ ^24^ ^]^ or alternatively a high FR concentration (20 and 20 µM, respectively) in the absence of “C” (“iFR^hi^”) (Figure 2a).^[^ ^34^ ^]^ Seeded MA‐, MM‐, PM‐ and MLL‐derived myoblasts gave rise to myotubes upon exposure to the Conv.Diff., iFR^hi^ or iFRC conditions at day 10, which expressed myosin heavy chain (MYHC), albeit less under Conv.Diff. (Figure 2b,c; Figure S2a,b). Remarkably, only upon exposure to iFR^hi^ or iFRC treatment the myotubes contracted, thereby indicating functionality, and the total myotube surface area was significantly larger compared with myotubes derived using the Conv.Diff. condition (Figure 2c,d; Figure S2b and Movies S1–S3, Supporting Information). Additionally, we performed quantitative Real‐Time PCR (qRT‐PCR) analysis of muscle differentiation genes after 4 and 7 days in the Conv.Diff., iFR^hi^ or iFRC conditions using 6 myoblast lines derived from the 4 muscle types (Table S2). We observed that certain genes (*MYH3*, *MYOG*) showed a similar expression pattern under all conditions, whereas certain genes (*MYH1*, *MYH2 and MYH4*) were more highly expressed in myoblasts subjected to the iFR^hi^ and notably iFRC conditions (Figure S2c, Supporting Information). These results suggest a unique differentiation program in multiple myoblast lines treated with iFR^hi^ and iFRC, as early as day 4 of differentiation.

Given the observed contractility and upregulation of unique muscle differentiation markers upon treatment with iFR^hi^ and iFRC, we set out to dissect the effect of iMPC medium versus small molecule treatment during the differentiation course. By using RNA‐Seq and LC‐MS, we documented that proliferation conditions (myoblast or iMPC media) clustered separately from the differentiation conditions, with further sub‐division based on the muscle type and treatment, with the iFR^hi^ and iFRC conditions separated from the Conv. Diff. condition at the transcriptomic and proteomic levels (Figure 2e; Figure S2d, Supporting Information). Although the two proliferation media conditions clustered together and displayed a very high correlation, we identified DEGs between the two conditions, documenting an upregulation of differentiation genes such as *MYOG*, *DES*, *MYH3*, *NCAM1*, *TNNI2* and *TNNT3* in myoblasts cultured in iMPC medium, suggesting myogenic commitment (Figure 2f; Figure S2e, Supporting Information). This observation was supported by scRNA‐Seq, which demonstrated a slight increase in the “CPGs” and “myocyte” populations in myoblasts cultured in the iMPC medium (Figure S2f–h, Supporting Information).

Next, we compared myoblasts that were cultured in iMPC medium to myoblasts that were subjected to iFR^hi^ or iFRC treatment for 10–15 days, time points which were characterized by increased myogenesis and abundancy of contractile myotubes and mononucleated cells under the iFR^hi^ or iFRC conditions (Figure 2b–d; Figure S2a,b, Supporting Information). The bulk RNA‐Seq analysis revealed a large number of upregulated differentiated skeletal muscle genes under the iFR^hi^ or iFRC conditions compared with the iMPC medium alone, including *MYH1*, *MYH* *2*, *MYH8*, *MYF6*, *MB*, *MYMK*, *TNNT3*, and more (Figure 2g). In accordance with this observation, a large cohort of upregulated genes were annotated with the GO terms “*Muscle contraction*”, “*Muscle organ development*” and “*Sarcomere organization*” under the iFR^hi^ and iFRC conditions compared to the iMPC medium (Figure S2i, Supporting Information). Furthermore, LC‐MS revealed DEPs that were significantly upregulated upon iFR^hi^ or iFRC treatment such as DES, MYH2, NEB, MYOM2 and other proteins (Figure S3a, Supporting Information). Last, upregulated DEGs and DEPs were annotated with various GO terms indicative of skeletal muscle tissue while downregulated DEGs and DEPs with GO terms indicative of reduced proliferation and DNA replication, among other terms (Figure S3b,c, Supporting Information).

As the next step, we integrated the transcriptome and proteome datasets and detected a concordance between the iFR^hi^ and iFRC conditions versus iMPC medium, demonstrating elevated expression of a large cohort of differentiation markers including mature skeletal muscle genes/proteins such as *MYOZ1*, *MYOZ2*, *CASQ1*, *MYH1, MYH4* and more (Figure 2h). Although the iFR^hi^ and iFRC conditions exhibited elevated expression of myogenic differentiation genes versus iMPC medium, when compared with each other, we identified subtle differences, most notably a higher expression of several key differentiation markers under the iFRC condition including *MYH2*, *MYH7*, *MYL3*, *TNNT1*, and more (Figure S3d, Supporting Information). In addition, GO terms indicative of skeletal muscle differentiation were enriched in the iFRC condition, whereas GO terms indicative of extracellular matrix (ECM) and metabolic processes were enriched in the iFR^hi^ condition, suggesting elevated myogenic differentiation under iFRC (Figure S3e,f, Supporting Information). In conclusion, our findings reveal that culturing myoblasts in iMPC medium resulted in a slight myogenic commitment, whereas the iFR^hi^ condition, and even more so the iFRC condition, induced prominent myogenic differentiation of myoblasts, characterized by upregulation of key muscle genes and proteins.

### 2.3 scRNA‐Seq Unveils Unique Small Molecule‐Derived Progenitor and Differentiated Cells

**Figure 3:**
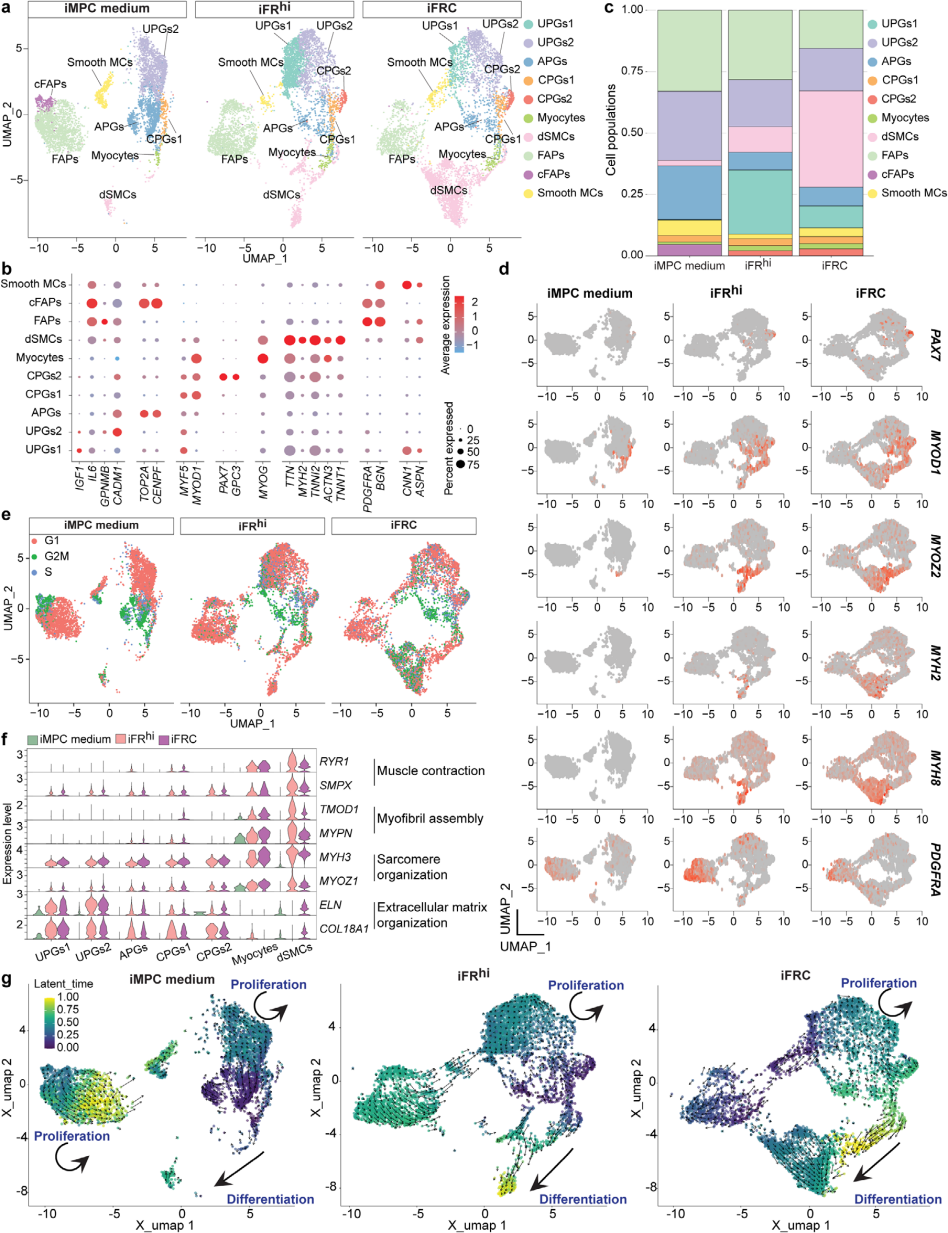
scRNA‐Seq reveals distinct populations in small molecule‐treated myoblasts. a) UMAP projection based on scRNA‐Seq of integrated cells from the indicated conditions colored by specific cell types. The cultures were analyzed at day 10 of iFR^hi^ or iFRC treatment. An MA myoblast line was used for the scRNA‐Seq analysis. b) Dot plot showing the expression level of representative marker genes in the indicated cell populations. c) Bar plots showing relative cell type distribution across the indicated cell cultures. Note cell populations that are highly enriched or solely detected under the iFR^hi^ and iFRC conditions. d) UMAP projection of integrated cells colored by the indicated marker gene expression. e) UMAP projection of integrated cells colored by cell cycle states. f) Violin plots showing expression of DEGs associated with the indicated GO terms in the specified cell populations that are highly expressed in the iFR^hi^ and iFRC conditions. g) UMAP projection of integrated cells colored by latent time as calculated by RNA velocity. Note enhanced gene expression dynamics in the iFR^hi^ and iFRC conditions, showing prominent progression toward myogenic differentiation.

Our findings thus far revealed that iFR^hi^ and iFRC treatment of bovine myoblasts resulted in substantial myogenic differentiation. However, the single cell composition and in particular which cell population/s may account for the prominent differentiation observed with small molecule treatment remained unknown. To investigate this question, we sought to characterize with scRNA‐Seq the various cell populations comprising MA‐derived myoblast cultures subjected either to the iMPC medium condition (Figure S2f–h, Supporting Information) or to iFR^hi^ and iFRC treatment. Through the analysis of 16,185 cells, we identified and annotated 10 cell populations in the three integrated samples including: *MYF5* ^+^/*IGF1* ^+^ UPGs1, *MYF5* ^+^/*CADM1* ^+^ UPGs2, *TOP2A* ^+^/*CENPF* ^+^ APGs, *MYF5* ^+^/*MYOD1* ^+^ CPGs1, *PAX7* ^+^/*MYOD1* ^+^ CPGs2, *MYOG* ^+^/*MYOD1* ^+^ myocytes, *TTN* ^+^/*MYH2* ^+^ dSMCs, *PDGFRA* ^+^ FAPs, *PDGFRA* ^+^/*TOP2A* ^+^ cFAPs and *CNN1* ^+^ Smooth MCs (**Figure** **3**a–e). Of note, while in iMPC medium the most prominent cell populations were UPGs2, APGs and FAPs/cFAPs, upon treatment with iFR^hi^ and iFRC, these cell populations reduced in proportions (Figure 3a,c). Furthermore, a few cell populations emerged only in iFR^hi^ and iFRC‐treated cultures including UPGs1, characterized by the expression of the progenitor marker *MYF5*, as well as CPGs2, characterized by the expression of the muscle stem cell marker *PAX7*,^[^ ^35^ ^]^ cautiously suggesting a de‐differentiation process and acquisition of a progenitor cell fate in these cell populations (Figure 3b,d). Moreover, the dSMC population was larger under the iFR^hi^ and iFRC conditions, exhibiting the expression of differentiation markers including *MYH2*, *MYH8*, *MYOZ2, TNNT1*, and more (Figure 3a–d).

Next, we performed a pathway enrichment analysis of DEGs based on scRNA‐Seq, unveiling the GO terms “*Muscle contraction*” and “*Sarcomere organization*” in myocytes and dSMCs, whereas “*Extracellular matrix organization*” was indicative of UPGs1 and UPGs2 (Figure 3f). Additionally, through RNA velocity analysis, we observed in iMPC medium enhanced temporal transcriptional dynamics in the FAP and UPG cell populations, however little to no progression toward dSMCs (Figure 3g). In contrast, in the iFR^hi^ and iFRC conditions, we observed prominent temporal transcriptional dynamics from CPGs to myocytes and dSMCs, indicating an active differentiation process (Figure 3g). Moreover, we noted progression from FAPs to UPGs, cautiously suggesting that compound treatment may involve a FAP‐to‐muscle progression, accounting for the reduced number of FAPs under iFRC (Figure 3g). In summary, the scRNA‐Seq analysis corroborated our previous findings, demonstrating enhanced myogenic differentiation under iFR^hi^ and iFRC treatment of myoblasts. Additionally, it enabled the dissection of various cell populations comprising the myogenic cultures, identifying progenitor‐like and differentiated cell populations unique to myoblasts subjected to small molecule treatments.

### 2.4 Improved Capture of Bovine Myogenesis In Vitro using Small Molecules

**Figure 4:**
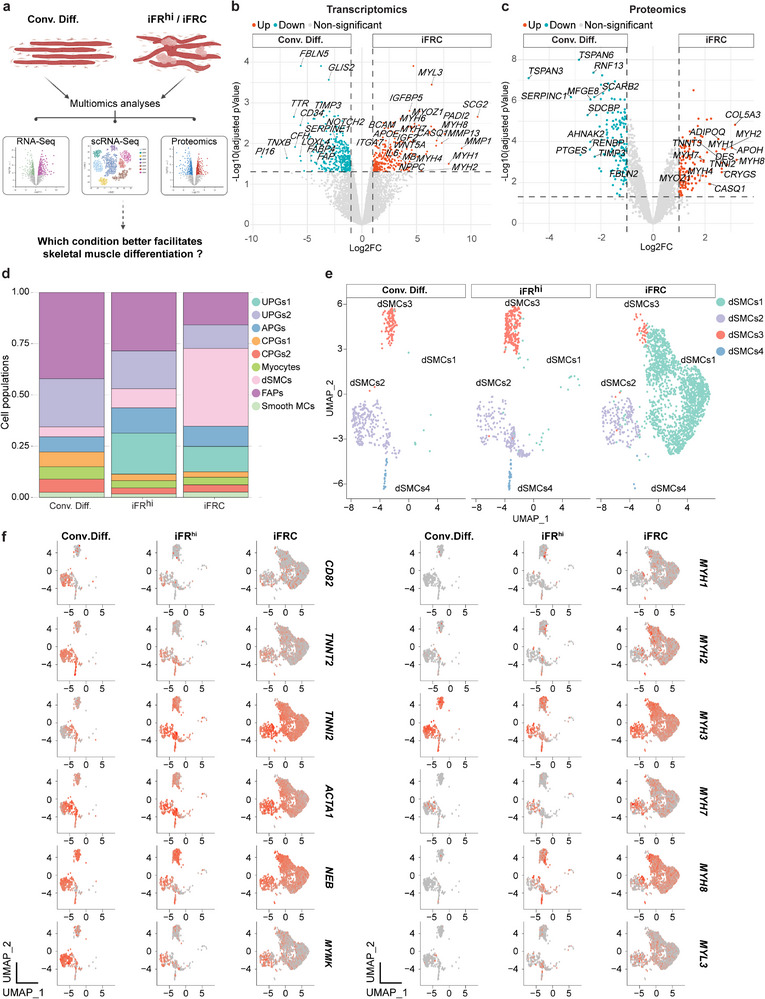
A unique myogenic program induced in myoblasts by small molecules. a) A schematic illustrating the experimental objective. b) Volcano plot based on RNA‐Seq showing DEGs between the indicated conditions. Significant DEGs (|log2FC| > 1, FDR < 0.05) are shown as colored dots. N = 3 myoblast lines per group, derived from three different muscle types. The cultures were analyzed at day 15. c) Volcano plot based on LC‐MS showing DEPs between the indicated conditions. Significant DEPs (|log2FC| > 1, FDR < 0.05) are shown as colored dots. N = 4 myoblast lines per group, derived from four different muscle types. The cultures were analyzed at day 10. d) Bar plots based on scRNA‐Seq showing relative cell type distribution in the indicated differentiation conditions. e) UMAP projection based on scRNA‐Seq of integrated dSMCs colored by specific sub‐population dSMCs1‐4. f) Split feature plots showing the expression level of the indicated skeletal muscle markers across dSMCs1‐4. Note the exclusive or substantial upregulation of several key muscle markers in the iFR^hi^ and iFRC conditions compared to the Conv.Diff. condition. The cultures were analyzed at day 10. An MA myoblast line was used for the scRNA‐Seq analysis in this figure. Note that some of the data presented in this figure were used for different objectives in Figures 1, 2, 3.

Our observations to this point revealed that iFR^hi^ and iFRC treatment of bovine myoblasts upregulated a myogenic differentiation program accompanied by the appearance of distinct cell populations. However, to what extent this myogenic differentiation program may differ from myotubes derived through a conventional method remained unexplored. To address this, we opted to directly compare the Conv.Diff. to the iFR^hi^ and iFRC conditions using the same multiomics datasets used before (Figures 1, 2, 3), that include bulk RNA‐Seq, scRNA‐Seq and LC‐MS (**Figure** **4**a). As a first step, differential gene and protein analyses revealed several muscle‐related DEGs (*MYOZ1*, *IGFBP5* and more) and DEPs (MYH8, TNNT3 and more) that were significantly upregulated in myoblasts subjected to the iFR^hi^ versus Conv.Diff. conditions (Figure S4a–d, Supporting Information). In contrast, myoblasts subjected to the Conv.Diff. condition did not exhibit a unique upregulation of muscle markers, suggesting that myogenic genes or proteins expressed in this culture are also expressed in iFR^hi^‐induced myogenic cells (Figure S4a–d, Supporting Information). Next, a comparison between the Conv.Diff. and iFRC conditions revealed DEGs (*MYH1*, *MYH2*, *MYH7*, *MYOZ1* and more) and DEPs (CASQ1, MYH2, MYH4, MYOZ1 and more) that were more highly expressed following iFRC treatment (Figure 4b,c; Figure S4e,f, Supporting Information). Collectively, in comparison with conventionally differentiated myoblasts, iFRC treatment gave rise to more DEGs and DEPs (648 and 263, respectively) than iFR^hi^ (461 and 219, respectively), suggesting increased myogenic differentiation in vitro.

To gain further insights into cellular dynamics during differentiation under the various conditions, we employed scRNA‐Seq to directly compare MA‐derived myoblasts subjected to the Conv.Diff., iFR^hi^ and iFRC conditions (7948, 5369 and 5300 cells, respectively, representing the same dataset shown in Figure 1 and Figure 3). Through integration of all three datasets, totaling 18,617 cells, we noted that iFR^hi^ and iFRC treatment of myoblasts resulted in a larger dSMC population, and uniquely contained the population UPGs1, whereas FAPs and UPGs2 decreased (Figure 4d; Figure S5a,b, Supporting Information). Interestingly, under the iFRC condition, CPGs2 showed elevated expression of *PAX7* (Figure S5c, Supporting Information). Furthermore, UPGs1 were characterized by *IGF1* and *WNT5A* expression, whereas certain genes, such as *PTN* and *SDC1*, were also expressed in UPGs2 (Figure S5d, Supporting Information). Furthermore, RNA velocity analysis for temporal transcriptional dynamics revealed a unidirectional differentiation trajectory in Conv.Diff. from myocytes to dSMCs (Figure S5e, Supporting Information). However, mostly under the iFR^hi^ and iFRC conditions, we identified transcriptional dynamics indicative of self‐renewal in UPGs and APGs, in addition to a bidirectionality in the myocytes and CPGs1 cell populations, that bifurcated toward dSMCs or CPGs2 and UPGs, cautiously suggesting a de‐differentiation process (Figure S5e, Supporting Information).

As the next step, we performed a functional annotation analysis and observed unique genes and their associated GO terms that were upregulated in the iFR^hi^ and iFRC conditions such as “*Muscle filament sliding*”, “*Muscle contraction*” and more (Figure S5f, Supporting Information). This and former observations prompted us to investigate the dSMC population in more detail, seeking to find differences between the three differentiation conditions. A re‐clustering and examination of dSMCs unveiled 4 dSMC sub‐populations (dSMCs1‐4), including dSMCs1, which was almost exclusively detected in the iFRC condition (Figure 4e; Figure S5g,h, Supporting Information). Furthermore, while all dSMC sub‐populations expressed various myogenic markers, dSMCs1 uniquely expressed several skeletal muscle‐related collagens (Figure S5i,j, Supporting Information).^[^ ^36^, ^37^ ^]^ We identified well‐known myogenic differentiation genes under all conditions including *TNNT2, TNNI2, ACTA1* and *NEB* (Figure 4f). Interestingly, among the *MYL* and *MYH* isoforms, the embryonic isoform (*MYH3*) was expressed in dSMCs under all three conditions, whereas other *MYL* or *MYH* isoforms (*MYL3*, *MYH1*, *MYH2* and *MYH8*) were almost exclusively expressed in iFR^hi^‐ and iFRC‐derived dSMCs, suggesting myogenic maturation and presence of both slow‐ and fast‐twitch muscle cells (Figure 4f). In conclusion, an in‐depth analysis of dSMCs at both the bulk and single‐cell levels revealed the partial acquisition of a myogenic differentiation program under the conventional differentiation protocol, and a more comprehensive capture of a myogenic program under iFR^hi^ and even more so the iFRC condition. Importantly, this analysis also revealed that small molecules promote differentiation into both oxidative slow‐twitch (*MYH7)*, oxidative fast‐twitch (*MYH2*) and glycolytic fast‐twitch (*MYH1*, *MYH4*) muscle cells in vitro.^[^ ^38^ ^]^

### 2.5 Small Molecules Generate Myogenic Cells In Vitro Resembling Muscle Cells In Vivo

**Figure 5:**
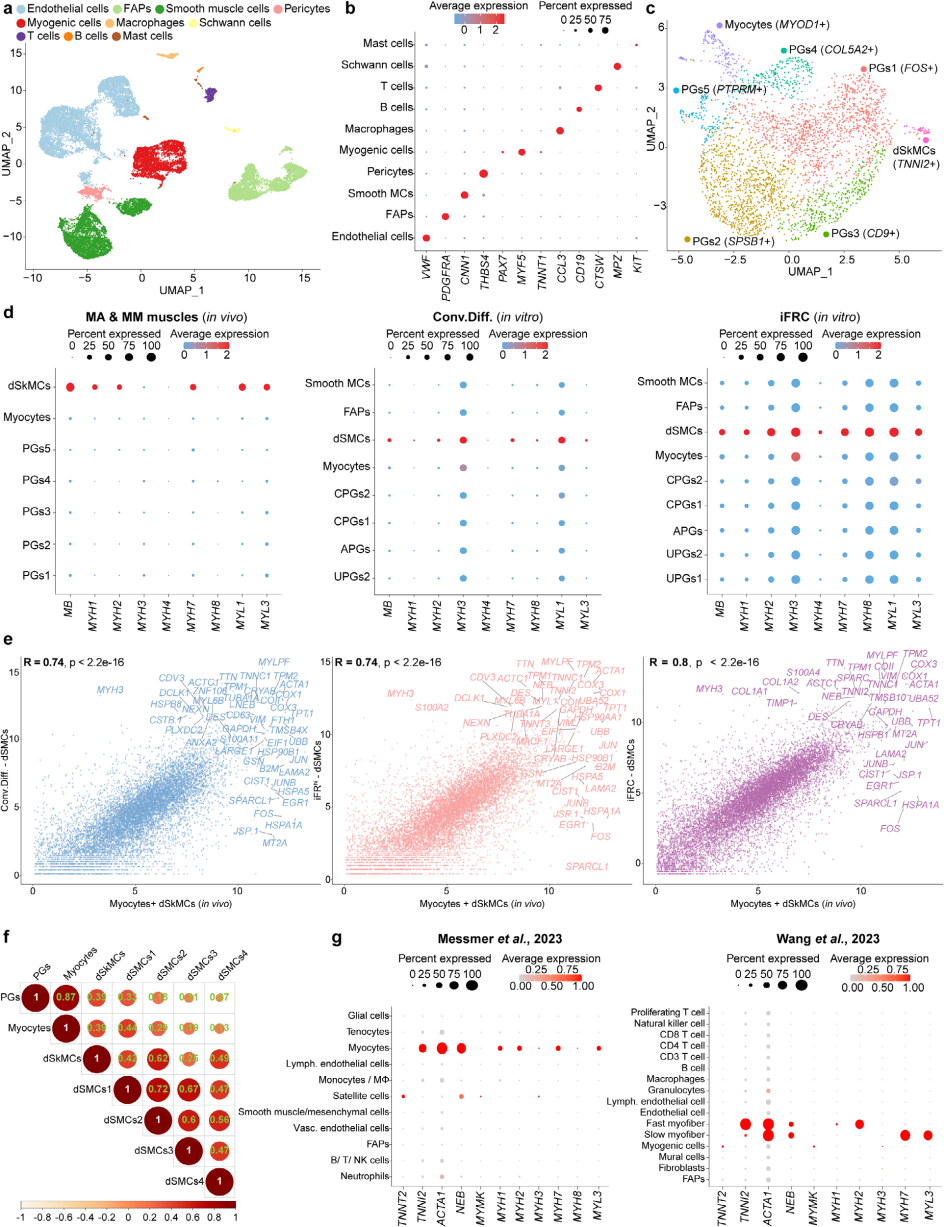
Small molecule‐derived muscle cells resemble skeletal muscle cells in vivo. a) UMAP projection based on scRNA‐Seq of in vivo‐derived bovine skeletal muscle tissue, generated through integration of MA and MM muscles, as colored by specific cell populations. b) Dot plot showing the expression level of marker genes indicative of the annotated cell populations. c) UMAP projection of integrated myogenic cells colored by the specific sub‐populations: Myogenic progenitors1‐5 (PGs1‐5), myocytes and differentiated skeletal muscle cells (dSkMCs). d) Dot plots demonstrating expression of the indicated myogenic genes from the specified in vitro‐derived cell types, in comparison to their expression in bovine muscle cells in vivo. The in vitro cultures were analyzed at day 10. An MA myoblast line was used for the scRNA‐Seq analysis. e) Scatter plots showing gene expression correlation between in vivo‐derived myocytes/dSkMCs and in vitro‐derived dSMCs1‐4, generated using the three indicated differentiation protocols. Pearson correlation coefficient (R) values and p‐values are provided. f) A plot showing Pearson correlation coefficient (R) values between in vivo‐derived dSkMCs and in vitro‐derived dSMCs1‐4, denoting integration of the three conditions shown in (e). g) Dot plots showing expression of the indicated myogenic genes in scRNA‐Seq datasets from in vivo‐derived bovine muscles generated by the specified studies.^[^ ^40^, ^41^ ^]^

A relevant question arising from the findings to this stage is whether the in vitro myogenic cultures resemble muscle cells in vivo. To explore this, we performed scRNA‐Seq of in vivo‐derived bovine MA and MM muscles, aiming to identify the myogenic cells, particularly differentiated skeletal muscle cells (dSkMCs), for comparison with in vitro‐derived dSMCs. By integrating the MA and MM scRNA‐Seq datasets (19,907 cells in total), we identified a variety of skeletal muscle resident cells including: *VWF^+^* endothelial cells, *CNN1^+^* smooth muscle cells (“Smooth MCs”), *PDGFRA^+^* FAPs, *MYF5^+^* myogenic cells, and more (**Figure** **5**a,b; Figure S6a, Supporting Information).^[^ ^39^, ^40^, ^41^, ^42^ ^]^ Within the myogenic cluster, we identified distinct cell populations including five *MYF5^+^* myogenic progenitors (PGs1‐5), *MYOD^+^*/*MYOG^+^* myocytes and a very small population of dSkMCs expressing a wide array of differentiation genes including *ACTA1*, *MYOZ1, MB, MYH2, TNNT3*, and more (Figure 5c; Figure S6b–d, Supporting Information). Of interest, we identified PGs1 as a satellite cell population associated with a stress response due to the expression of *FOS* and *ATF3* (Figure S6b,d, Supporting Information).^[^ ^43^ ^]^ In addition, PGs3 and PGs5 expressed *PAX7*, confirming this gene is a bovine muscle stem cell marker (Figure S6a,c, Supporting Information). Finally, we could not detect distinct sub‐populations within the myocytes or dSkMCs, most likely as scRNA‐Seq only captures a small number of mononucleated cells and not myonuclei in myofibers (Figure 5c; Figure S6a–d, Supporting Information).

As a next exploratory step, we set out to compare the expression of myogenic differentiation genes captured in myocytes and dSkMCs in vivo, to the in vitro‐derived dSMCs (scRNA‐Seq datasets shown in Figures 1, 3, 4). Notably, we detected several genes which were expressed in vivo, as well as within dSMCs obtained using the Conv.Diff. or iFRC conditions including *MYL1*, *ACTA1*, *NEB*, and more (Figure 5d; Figure S6e, Supporting Information). Remarkably, we detected a higher expression of several differentiation genes preferentially in dSkMCs and iFRC‐derived dSMCs, including *MYH1*, *MYH* *2*, *MYH* *7*, *MYL3* and more, whereas a few genes were detected almost solely in iFRC‐derived dSMCs (*MYH4, MYH8*) (Figure 5d). These results are in accordance with the former observation that iFR^hi^ and iFRC treatment of myoblasts elicits upregulation of unique myogenic differentiation genes (Figure 4).

Building upon this observation, we performed a correlation analysis between the in vivo‐derived myocytes and dSkMCs, as well as the in vitro‐derived dSMC sub‐populations. This comparison revealed a higher correlation between iFRC‐derived dSMCs and in vivo‐derived dSkMCs (0.8), compared to the Conv.Diff.‐ and iFR^hi^‐derived dSMCs (0.74 and 0.74, respectively) (Figure 5e). Next, we performed an in‐depth correlation analysis between the in vivo‐derived PGs, myocytes and dSkMCs to the in vitro sub‐populations dSMCs1‐4. This analysis revealed improved correlation between dSMCs1, PGs and myocytes, whereas dSMCs2 showed the highest correlation with dSkMCs, albeit all correlations were not high (Figure 5f). As dSMCs1 appeared exclusively in the iFRC condition, we postulate that this sub‐population contributed to the resemblance of iFRC‐derived dSMCs to dSkMCs. Finally, we analyzed scRNA‐Seq datasets of bovine muscles generated by other groups,^[^ ^40^, ^41^ ^]^ identifying myocytes and dSkMCs expressing myogenic genes such as *MYL3*, *MYH2*, *MYH7* and more (Figure 5g). Importantly, we detected a higher expression of these myogenic markers in iFRC‐derived dSMCs versus Conv.Diff.‐derived dSMCs, thus supporting our findings (Figure 5d). In conclusion, several key muscle genes were exclusively expressed in MA and MM muscles in vivo and in myoblasts treated with iFRC in vitro, leading to iFRC‐ derived dSMCs closely resembling bovine muscle cells in vivo.

### 2.6 Rapid and Mature Skeletal Muscle Formation in 3D Constructs using Small Molecules

**Figure 6:**
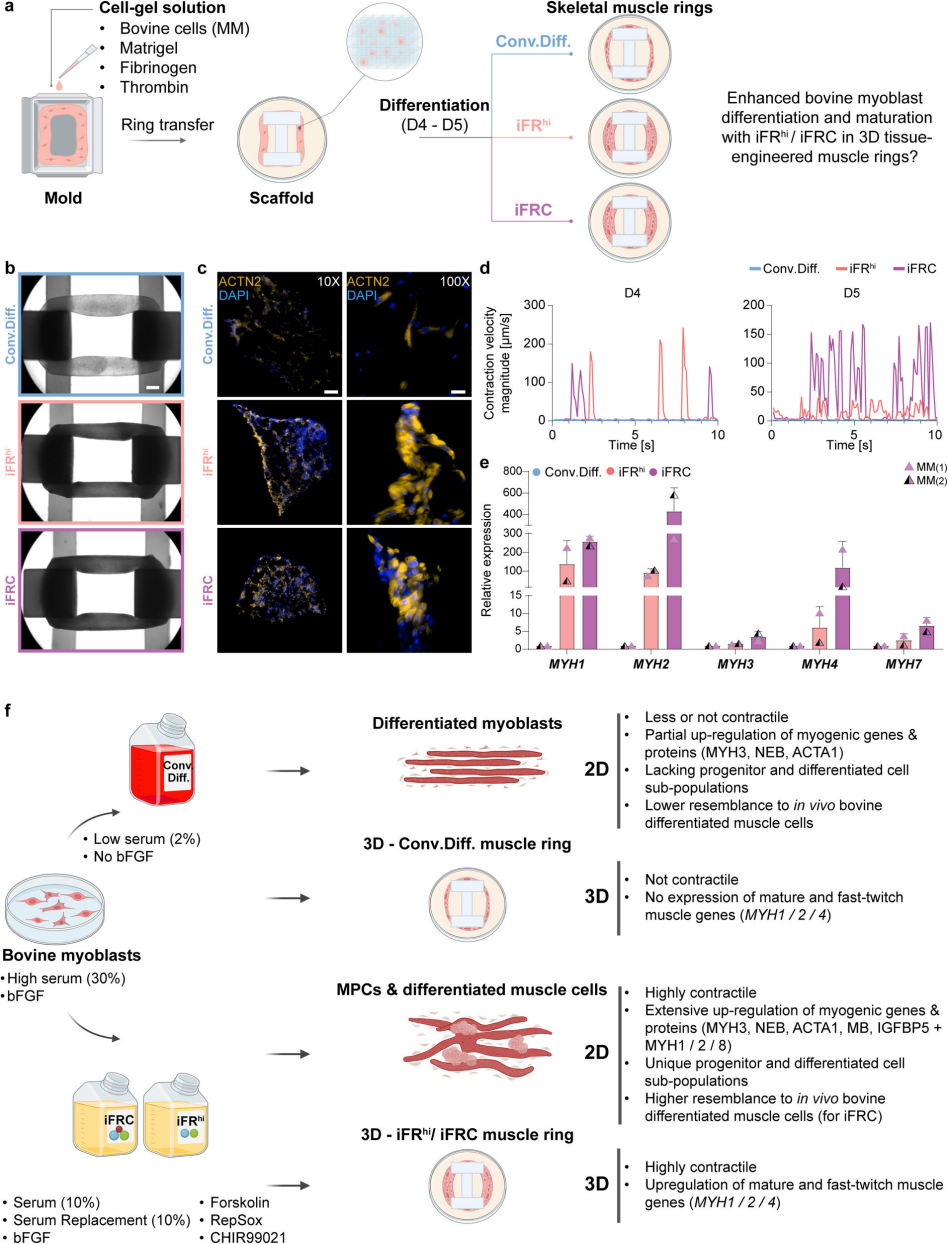
Enhanced myogenic differentiation in 3D constructs with small molecules. a) A schematic outlining the tissue engineering strategy employed to investigate whether iFR^hi^ and iFRC conditions facilitate bovine myoblast differentiation in 3D tissue‐engineered muscle rings. b) Representative phase contrast images depicting 3D bovine muscle rings generated using the specified conditions at day 5. LUTs were equally adjusted. Scale bar, 1000 µm. c) Representative immunofluorescence images of muscle ring cross‐sections generated using the indicated conditions at day 5 of differentiation. Nuclei were stained for DAPI and cells were stained for the myogenic differentiation marker ACTN2. Note that the images in the right panel are higher‐magnification views of those in the left panel. LUTs were equally adjusted. Scale bar, 200 µm (left panel) and scale bar, 20 µm (right panel). d) A graph showing measurement of contraction displacement for MM‐1 myoblast‐derived muscle rings across time at day 4 and 5. N = 1 MM myoblast line. Note that in this experiment, only muscle rings derived using small molecules exhibited contractility. e) qRT‐PCR analysis of the indicated myogenic differentiation genes in bovine muscle rings generated using the specified differentiation conditions. Note increased expression of fast‐twitch and glycolytic *MYH* isoforms with iFR^hi^ and iFRC treatment. N = 2 different MM myoblast lines. Error bars denote standard deviation (SD). f) Summary of the study results.

Enhanced differentiation with the iFR^hi^ and iFRC conditions has so far been observed in 2D cultures. However, propagation and differentiation of muscle cells for cultivated meat purposes is expected to occur in a 3D format to achieve a substantial biomass or within tissue‐like aligned structures.^[^ ^44^ ^]^ We consequently wished to assess the effect of small molecules on the differentiation and maturation of bovine myoblasts during the generation of 3D tissue‐engineered muscle constructs. To this end, we tested whether the myoblasts could form skeletal muscle rings through casting into designated molds, followed by differentiation in printed scaffolds, which enable anisotropic tissue formation and maturation (**Figure** **6**a).^[^ ^45^ ^]^ To enhance cell viability and differentiation, we embedded MM‐derived myoblasts in a biomaterial solution consisting of Fibrinogen, Thrombin and Matrigel.^[^ ^45^ ^]^ The myoblast‐containing solution was then injected into the molds. After 24 h, skeletal muscle rings were transferred to the scaffolds and cultured in myoblast medium for additional 48 h before switching to either the Conv.Diff., iFR^hi^ or iFRC conditions for 5 days of differentiation (Figure 6a). This effort resulted in the formation of 3D differentiated skeletal muscle rings, which appeared more condensed with the iFR^hi^ and iFRC conditions (Figure 6b). Sectioning and immunostaining of these 3D ring structures revealed widespread expression of the muscle differentiation marker ACTN2, both inside and in the periphery of the 3D muscle structures (Figure 6c; Figure S7a, Supporting Information). Remarkably, at day 4 and day 5 of differentiation, we also observed prominent contractility solely in bovine muscle rings produced with the iFR^hi^ or iFRC conditions, supporting previous observations in 2D cultures demonstrating the contractility of myotubes generated with small molecules, but not with the Conv.Diff. condition (Figure 6d; Figure S7b and Movies S4–S6, Supporting Information).

To investigate whether these morphological and functional attributes were accompanied by molecular characteristics, we performed qRT‐PCR for notable myogenic differentiation genes. Consistent with our previous data in 2D cultures, in comparison to the Conv.Diff. condition, we documented an upregulation of *MYH1, MYH2*, and *MYH4* in muscle rings generated with the iFR^hi^, and even more so the iFRC condition, whereas *MYH3* and *MYH7* expression was only slightly higher (Figure 6e). The upregulation of oxidative fast‐twitch (*MYH2*) and glycolytic fast‐twitch (*MYH1, MYH4)* genes with small molecule treatment may contribute to the contractility observed under the iFR^hi^ and iFRC conditions. Taken together, these data demonstrate that small molecules promote myoblast differentiation in skeletal muscle ring constructs, facilitating tissue maturation and functionality in a 3D model.

## 3 Discussion

In this study, we first employed multiomics to characterize bovine skeletal muscle differentiation in vitro using a conventional protocol. We then investigated the combined effect of small molecules affecting the cAMP, TGF‐β and WNT signaling pathways to improve the differentiation of bovine skeletal muscle cells in vitro in 2D and 3D formats. Our findings demonstrate the efficacy of the small molecules to give rise to myogenic progenitor‐like cells, while simultaneously promoting differentiation into multinucleated myotubes (Figure 6f). Importantly, in comparison with the conventional protocol for myoblast differentiation, the enhanced media gave rise to larger and contractile myotubes, expressing mature skeletal muscle markers, and demonstrating a closer resemblance to muscle cells in vivo (Figure 6f). These media conditions facilitated the formation of contractile 3D‐engineered muscle rings, which express myogenic genes associated with fast‐twitch and glycolytic myotubes, unlike muscle rings generated with a conventional protocol (Figure 6f).

Generation of cultivated meat products entails a multi‐step process with several potential bottlenecks for affordable and efficient scale‐up. Several notable challenges include the loss of proliferative capacity of MPCs in vitro, as well as an incomplete induction of myogenic properties and diminished differentiation capacity over successive passages.^[^ ^44^, ^46^ ^]^ To bridge this gap, in recent years new studies emerged that have begun characterizing bovine myogenesis in vitro, exploring its potential augmentation through modulation of signaling pathways.^[^ ^14^, ^47^, ^48^, ^49^ ^]^ For example, p38 inhibition was shown to prolong the stemness of bovine satellite cells in vitro while maintaining their myogenic potential over time.^[^ ^50^ ^]^ Moreover, individual or combined administration of small molecules affecting the MEK/ERK, NOTCH and RXR pathways enhanced the fusion capacity of bovine myoblasts in vitro.^[^ ^51^ ^]^ Recent research has also investigated the capacity of myoblasts to proliferate and differentiate in the absence of unfavorable animal‐based serum, which is a key aspect for in vitro meat production.^[^ ^52^, ^53^, ^54^ ^]^ As the iFR^hi^ and iFRC conditions contain 10% animal serum, it will be of interest to assess whether the compounds’ effect can enhance myogenesis and promote differentiation using serum‐free conditions in 2D and 3D models.

Our study provides, to our knowledge, one of the most comprehensive multiomics analyses of in vitro bovine myogenesis to date, integrating 15 RNA‐Seq and 18 proteomics samples, further encompassing a total of 47,253 single‐sequenced cells comprised of 27,346 in vitro‐derived cells and 19,907 in vivo‐derived cells. We believe this dataset will serve as a useful resource for studying bovine myogenesis, complementing recent works.^[^ ^40^, ^41^, ^42^, ^55^ ^]^ Surprisingly, the scRNA‐Seq analysis identified dSMCs expressing differentiated muscle genes, suggesting they may represent mononucleated myocytes before fusion. However, it is also noteworthy to mention that through scRNA‐Seq, we only analyzed the content of the mononucleated cells within 2D cultures, but not myonuclei within myotubes. Therefore, it will be of interest to utilize single‐nucleus RNA‐Seq (snRNA‐Seq) to assess whether the expression of distinct muscle genes is replicated in myonuclei derived using iFR^hi^ or iFRC treatment, compared to the conventional protocol.

Treating myoblasts with iFR^hi^ or iFRC induced myogenic differentiation within 4–15 days across multiple myoblast lines isolated from different muscle types and cows, which we consider advantageous for broader applicability. Notably, however, we did not investigate the capacity of the treated myoblasts to proliferate over multiple passages and maintain myogenic attributes, a trait that will be of interest for cultivated meat applications, as myoblasts tend to lose differentiation potential with prolonged passaging thereby requiring immortalization.^[^ ^28^ ^]^ In this context, it will be of interest to assess whether myoblasts that have lost their potential to differentiate into myotubes under conventional conditions, or alternatively immortalized myoblasts, may increase their myogenic differentiation potential following supplementation with iFR^hi^ or iFRC. Additionally, the molecular mechanisms underlying how the small molecules induce a myogenic differentiation program in bovine myoblasts remains to be explored. In particular, as the small molecule downstream targets are known, it will be of interest to assess whether overexpression of such targets can replace specific small molecule treatment. For example, overexpression of *CREB1*, which is upregulated by Forskolin,^[^ ^56^ ^]^ or β‐catenin, which is induced upon GSK3 inhibition,^[^ ^57^ ^]^ may mimic the effects of the respective compounds. Additionally, it will be of interest to assess if myogenic genes are rapidly demethylated in iMPC medium, which has serum replacement containing ascorbate, a co‐factor for TET enzymes.^[^ ^58^ ^]^

For dietary purposes, the accurate capture of muscle cells in vitro that express a wide repertoire of muscle genes and proteins may prove beneficial due to enhanced nutritional value. This notion is especially important when compared with the conventional myoblast differentiation protocol, in which the expression of key muscle proteins was undetected, particularly fast‐twitch and glycolytic fiber type markers, that were detected in muscles in vivo and in iFRC‐derived dSMCs. This aspect may be of interest for the generation of steaks and filets, which unlike minced meat products, represent an organized muscle tissue composed of multiple cell and fiber types, posing greater challenges to replicate, and necessitating the addition of adipocytes and fibroblasts for flavor and texture.^[^ ^30^, ^59^, ^60^ ^]^ Recent studies have shown the generation of such tissue‐like structures resembling meat products through tissue engineering applications.^[^ ^61^, ^62^, ^63^, ^64^, ^65^, ^66^, ^67^, ^68^ ^]^ As former studies usually employed conventional differentiation protocols, we envision that a short administration of iFRC during myoblast differentiation, as exemplified in the 3D muscle rings in this study, may lead to a tissue maturity that more closely resembles meat, offering increased protein content and nutritional value. In closing, although extensive additional work is required, we believe that the approach outlined in this study may contribute a valuable step toward achieving the goal of replacing conventionally‐derived meat with more environmentally friendly and ethically sustainable alternatives.

## 4 Experimental Section

### Generation of Myoblast Cell Lines from Bovine Muscles

Bovine muscle samples from *Musculus obliquus externus abdominis* (MA), *musculus masseter* (MM), *musculus psoas major* (PM) and *musculus longissimus lumborum* (MLL) were taken, up to 48 h after sacrifice, from the Zurich or Hinwil slaughterhouses (Switzerland). Small muscle fragments were then transported on ice to the laboratory at ETH Zurich for processing. Satellite cells were isolated on the same day to establish primary myoblast cultures. For this, muscle samples were cleaned in phosphate‐buffered saline (PBS, 10010015, Thermo Fisher Scientific), Braunol (18392, B.Braun) and 70% ethanol. The muscle tissue was then minced and incubated in the first digest solution consisting of Dulbecco`s Modified Eagle Medium (DMEM, 41966052, Thermo Fisher Scientific) supplemented with 0.2% Collagenase Type II (17101015, Thermo Fisher Scientific) for 1 – 1.5 h at 37 °C in a shaking water bath. The cell suspension was washed with a “wash buffer” composed of Ham`s F‐10 Nutrient Mixture (F‐10, 22390025, Thermo Fisher Scientific) supplemented with 10% horse serum (HS) (16050122, Thermo Fisher Scientific). This was followed by a second digest with 0.4% Dispase II (17105041, Thermo Fisher Scientific) diluted in PBS. The cell suspension was vortexed and incubated for 30 min at 37 °C in a shaking water bath. The cells were then triturated, filtered and washed before the final cell pellet was resuspended in “myoblast medium” consisting of 1:1 DMEM and F‐10 supplemented with 20% fetal bovine serum (FBS, A5256701, Thermo Fisher Scientific), 10% HS, 1% penicillin‐streptomycin (Pen‐Strep) (15‐140‐148, Thermo Fisher Scientific) and bFGF (10 ng mL^−1^, 223‐FB, R&D Systems). The myoblasts were derived using a pre‐plating technique to remove fibroblasts and other adherent cells, leaving non‐adherent cells such as satellite cells in suspension for up to 4 h before the supernatant was collected and transferred onto Matrigel (354234, Corning) coated plates. Cells were cultured in myoblast medium with Normocin (ant‐nr‐1, InvivoGen) (1:500) for several days after isolation and tested negative for mycoplasma (MycoStripTM 100, rep‐mys‐10, InvivoGen).

### Myoblast Expansion

All myoblasts used in this study were between passages 1–4. Myoblasts were propagated using plates coated with a solution of 4% Matrigel (354234, Corning) in low glucose DMEM (31885049, Thermo Fisher Scientific). For this, Matrigel coated plates were placed on ice for 7 min. Then, the Matrigel solution was collected, and the plates were incubated for 1 h at 37 °C. Where indicated, myoblasts were cultured in “iMPC medium”^[^ ^24^ ^]^ consisting of KnockOut DMEM (10829018, Thermo Fisher Scientific), supplemented with 10% FBS, 10% KnockOut Serum Replacement (10828028, Thermo Fisher Scientific), 1% GlutaMAX (35‐050‐061,Thermo Fisher Scientific), 1% MEM non‐essential amino acid solution (11140050, Thermo Fisher Scientific), 1% Pen‐Strep, 0.1% 2‐mercaptoethanol and bFGF (10 ng mL^−1^). Typically, myoblasts subjected to proliferation or differentiation media (i.e., Conv.Diff., iFR^hi^ and iFRC conditions) were seeded onto plastic culture plates without Matrigel coating.

### Myoblast Differentiation

Approximately 1 × 10^5^ myoblasts were seeded onto a 6‐well plate on “Day ‐1” in myoblast medium. On “Day 0”, the cells were cultured in a respective differentiation medium. For the conditions “iFR^hi^” and “iFRC”, myoblasts were cultured in these conditions from Day 0 onwards. Furthermore, for the “iFR^hi^” medium, 20 µM Forskolin (1099/50, R&D Systems) and 20 µM RepSox (3742/50, R&D Systems) were supplemented at Day 0. For the condition “iFRC” medium, 5 µM of Forskolin, 5 µM RepSox and 3 µM CHIR99021 (4423/50, R&D Systems) were supplemented from Day 0. To initiate conventional myoblast differentiation, myoblast medium was replaced with “Conv. Diff.” medium composed of DMEM supplemented with 2% HS and 1% Pen‐Strep on Day 4 of cell seeding, and the cultures were then subjected to the Conv. Diff. condition for an additional 6–11 days before analysis, depending on the specified experiment. For the timepoint analysis via qRT‐PCR, RNA was isolated from all cell cultures after 4 or 7 days of differentiation (see also the “qRT‐PCR” method section).

### Microscopy, Immunofluorescence Staining and Image Analysis

Cells were fixed with 4% paraformaldehyde (PFA, 11400580, Thermo Fisher Scientific) for 5 min at room temperature (RT) and washed with PBS. A blocking solution consisting of PBS supplemented with 2% bovine serum albumin (BSA) (9048‐46‐8, AppliChem), 1% Triton X‐100 (X100‐100ML, Sigma–Aldrich) was added to the cells for 1 h at room temperature (RT). Then, a staining solution consisting of PBS supplemented with 2% BSA, with the respective primary antibodies was added to the cells overnight at 4 °C. After incubation, cells were washed twice and stained with secondary antibodies in staining solution supplemented with 1:1000 4′,6‐ diamidino‐2‐phenylindole (DAPI, 62248, Thermo Fisher Scientific) for 1 h at RT. The following primary antibodies were used for 2D myogenic cultures: anti‐mouse/rat/human myogenin (MYOG, 1:1000, SC‐12732, Santa Cruz Biotechnology), anti‐mouse/rat/human myosin heavy chain (MYHC, 1:500, MAB4470, R&D Systems). The following secondary antibodies were used: Alexa Fluor 647 goat anti‐mouse IgG1 (A21240, Thermo Fisher Scientific) and Alexa Fluor 488 goat anti‐mouse IgG2B (A21141, Thermo Fisher Scientific), both at a 1:400 dilution. Phase contrast or immunofluorescence images were taken with a Nikon ECLIPSE Ti2 microscope. Look up tables (LUTs) were equally adjusted unless indicated otherwise. In certain cases, due to differences in cell density, or to improve signal clarity, LUTs were individually adjusted, as mentioned in the respective figure legends.

### Muscle Ring Sectioning and Immunofluorescence

The rings were removed from their scaffolds, washed with PBS and fixed in 4% PFA for 24 h at 4 °C, followed by a 24 h‐incubation period in a 30% sucrose solution at 4 °C. Fixed scaffolds were embedded using optimal cutting temperature (OCT) reagent (KMA‐0100‐00A, Cellpath) and stored at −80 °C. A 10 µm thick cryo‐sections were made using a cryostat (Leica CM1950) and stored at −80 °C. For immunofluorescence, sections were washed with PBS, blocked for 1 h at RT, followed by incubation with a primary antibody solution for 1 h at RT, washed, and then added a secondary antibody solution for 1 h at RT. Sections were then washed with PBS and mounted with ProLong Glass Antifade Mountant (P36984, Thermo Fisher Scientific). The following primary antibody was used: anti‐mouse ACTN2 (1:800 dilution, A7811, Sigma Aldrich) and the corresponding secondary antibody: Alexa Fluor 546 goat anti‐mouse IgG1 (1:400 dilution, A21123, Thermo Fisher Scientific). All images were taken using an ECLIPSE Ti2 microscope (Nikon).

### Myotube Surface Area Quantification

Myotube surface area quantification is based on the percentage area of an image occupied by myotubes as measured by immunofluorescence for MYHC. This analysis was calculated using four randomly chosen images per cell line and condition utilizing the MyoCount software^[^ ^70^ ^]^ and were also manually overseen to exclude false positive images. Bar plots were plotted using the GraphPad's Prism software version 10.

### Reverse Transcription and Quantitative Real‐Time PCR (qRT‐PCR)

Total RNA was isolated using an RNeasy mini (74106, Qiagen) or micro (74004, Qiagen; 3D scaffolds) kit following the manufacturer's instructions. The RNA concentrations were measured by a plate reader (Tecan). To obtain cDNA, the instructions of the High‐Capacity cDNA Reverse Transcriptase Kit (4368813, Thermo Fisher Scientific) were followed. The qRT‐PCR mix contained 10 ng cDNA, forward and reverse primers (10 µM) and Fast SYBR Green Master Mix (4385612, Thermo Fisher Scientific). The bovine *TBP* gene was used as a housekeeping gene. The condition “Conv.Diff.” was used as the control reference for analysis. The sequences of the primers used were listed in **Table** **1**. Primers were ordered and produced by Microsynth (Balgach, Switzerland). The qRT‐PCR was run on a QuantStudio 5 machine (Thermo Fisher Scientific). For each biological sample three technical replicates were used to obtain the CT values. In rare cases, where the target gene CT value was not detected, missing values were imputed by an arbitrary CT value of 40 for further calculations. Bar plots were plotted using GraphPad's Prism software version 10.

**Table 1:** Primer sequences used for qRT‐PCR.

Target gene	Sequence 5′ → 3′
*MYH1* – forward	TTGCATCTCCAAGGCAGGGTC
*MYH1* – reverse	CGAAAGGCTTATTCTGGGCCT
*MYH2* – forward	GGCTGACTCGTCCTGCTTTA
*MYH2* – reverse	GCTGAACTCAGAGGTCCTTGTT
*MYH3* – forward	AGAGCTGCTGCTCATCACAA
*MYH3* – reverse	GATGTCAATGGCGCTATCCG
*MYH4* – forward	GGTCCAAGTGCTGAAGAGGGT
*MYH4* – reverse	AGCGTACAAAGTGGGGGTG
*MYH7* – forward	CAAGTCCGCCTACCTCATGG
*MYH7*– reverse	TTTGGCATACACCACCTGCT
*MYOG* ^[^ ^50^ ^]^ – forward	GCGCAGACTCAAGAAGGTGA
*MYOG* ^[^ ^50^ ^]^ – reverse	TGCAGGCGCTCTATGTACTG
*TBP* – forward	AAGCGTTTTGCTGCTGTAATCA
*TBP* – reverse	CCATCTTCCCAGAACTGAATATCA

### Bulk RNA‐Seq

Total RNA was extracted from myoblasts 2 days after plating in myoblast proliferation medium, to prevent fusion and myotube generation, while RNA extraction from differentiated myoblasts was done at day 15. For this, an RNeasy mini kit was used according to the manufacturer`s instructions (74106, Qiagen). The RNA quality was assessed with a Fragment Analyzer (Agilent, Santa Clara, California, USA). The TruSeq Stranded mRNA kit (Illumina, Inc, California, USA) was used for library preparation, as previously described.^[^ ^71^ ^]^ All samples were sequenced in a single‐end 100 bp configuration on an Illumina NovaSeq 6000 at the Functional Genomics Center Zurich (FGCZ, Zurich, Switzerland). The raw sequencing reads were processed and analyzed using the SUSHI framework developed by FGCZ.^[^ ^72^ ^]^ For quality control, adapter sequences and low‐quality bases were removed using fastp v0.20^[^ ^73^ ^]^ and the resulting cleaned reads were pseudo‐aligned to the bovine reference genome assembly Bos_taurus_UMD_3.1 (Ensembl release v92) and gene expression levels were quantified using Kallisto v0.46.1.^[^ ^74^ ^]^ Differential gene expression analysis between various conditions was subsequently carried out with the R package edgeR v3.38.1^[^ ^75^ ^]^ by including tissue effect as a covariate, if required. Genes showing at least significant (FDR < 0.05) two‐fold change were classified as differentially expressed. Nominal p < 0.01 was used instead of FDR to determine significance for the iFRC versus iFR^hi^ conditions and iMPC versus myoblast media comparison.

### Proteomics with Tandem Mass Tag (TMT) Labeling

Myoblasts 2 days after splitting and myoblasts that were subjected to differentiation conditions on day 10 were thoroughly washed with PBS and then scrapped from the plates using a cell scraper. The respective cell aggregates were collected and centrifuged at 300 × g for 5 min followed by aspiration of the supernatant. Cell pellets were then snap frozen on dry ice for sample digestion and clean up. Then, cell pellets were lysed in 100 µL 4% SDS/Tris‐HCl and subjected to High Intensity Focused Ultrasound (HIFU) for two times for 1 min at an ultrasonic amplitude of 80%, followed by boiling at 95 °C for 10 min. Cell debris and other insoluble materials were removed by centrifugation at 20 000 × g for 10 min. Insoluble cell debris and other undesired components were separated and discarded through centrifugation at 20 000 × g for 10 min. The protein concentration was then assessed using the Lunatic UV/Vis polychromatic spectrophotometer (Unchained Labs). Protein samples were processed, including protein digestion, clean‐up and bead conditioning as previously described,^[^ ^76^ ^]^ with the following specifications: For each sample, 25 µg of protein was treated with 2 mM DTT (Dithiothreitol) at RT for 30 min to facilitate reduction, which was followed by alkylation using 15 mM IAA (Iodoacetamide) at 30 °C for 30 min protected from light. After merging the digest solution with the water elution, the mixture was fully dried.

### TMT Labelling and Peptide Fractionation

TMT labeling and peptide fractionation were performed as previously described,^[^ ^77^ ^]^ with the following modifications: a TMTpro 18‐plex reagent was employed, 10 µg of peptides were used for labeling, peptide separation was conducted on an XBridge Peptide BEH C18 column (4.6 mm × 250 mm), and the fractions were concatenated into eight final fractions.

### LC‐MS/MS Analysis

LC‐MS/MS analysis was performed as previously described^[^ ^77^ ^]^ with the following modifications: the normalized collision energy for HCD was adjusted to 32% with turboTMT enabled, and charge state screening rejects charge states higher than six. The local laboratory information management system (LIMS) was used to process the mass spectrometry data.^[^ ^78^ ^]^ The mass spectrometry run was limited to only 18 samples in a single batch due to the instrument capacity at the sequencing facility, accounting for the 18 samples used and reported in this study.

### Proteomics Data Analysis

Proteomics data analysis was performed as previously described,^[^ ^77^ ^]^ with the following modifications by using the UniProt bovine reference proteome (reviewed canonical version from 2022‐04‐22), and TMTpro modifications were applied to peptide N‐termini and lysine side chains. For each TMT label, FragPipe‐generated peptide spectrum match abundances (PSM) were filtered based on purity threshold = 0.5 and PeptideProphetProb = 0.9. Protein abundances were then calculated by aggregating the PSM abundances to peptidoform abundances applying Tukey's‐Median Polish algorithm. The estimated protein abundances were transformed using the variance stabilizing normalization^[^ ^79^ ^]^ prior to linear model fitting. Proteins with at least significant (FDR < 0.05) two‐fold change were considered to be differentially expressed.

### Integrative Bulk RNA‐seq/LC‐MS Analysis

For each conditional comparison, an integrated data set was assembled with the overlapping differentially expressed genes (DEGs) and differentially expressed proteins (DEPs) using their Ensembl identifiers. A Pearson correlation coefficient was calculated using their log2 fold change (log2FC) values. Data normality was assessed using Kolmogorov‐Smirnov test. The R package enrichR v3.2^[^ ^80^ ^]^ based on human WikiPathways database^[^ ^81^ ^]^ was used to perform functional enrichment analysis of DEGs and DEPs.

### scRNA‐Seq Sequencing

Myoblasts 2 days after splitting, or differentiated myogenic cultures at day 10, were washed with PBS and trypsinized (Trypsin‐EDTA 0.25%, 25200056, Thermo Fisher Scientific) for 5 min at 37 °C. Cells were filtered using a 40 µm cell strainer and pelleted at 300 g for 5 min. After a final wash with PBS, cells were counted using a Neubauer hemocytometer. To obtain the number of viable cells, the cell suspension was stained with Trypan Blue (T8154, Sigma–Aldrich). Next, cells were processed according to the manufacturer's Chromium Next GEM Single cell 3′ v3.1 protocol using the 10X Genomics platform. For muscle tissue isolation, the tissue was cleaned using Braunol (18392, BBraun), 70% ethanol and PBS. Then, the tissue was minced and enzymatically digested in a solution consisting of Hanks' Balanced Salt Solution (HBSS, 14025050, Thermo Fisher Scientific) supplemented with 0.2% Collagenase Type II (17101015, Thermo Fisher Scientific) and 1.5% Albumin (BSA) Fraction V (A1391,0100, AppliChem GmbH) for 1–1.5 h at 37 °C in a shaking water bath. The reaction was stopped by adding a medium consisting of low glucose DMEM (31885023, Thermo Fisher Scientific) supplemented with 10% FBS, followed by a filtration step using a 100 µm cell strainer. To deplete the sample of red blood cells, the pellet was incubated with ACK lysis buffer (A1049201, Thermo Fisher Scientific) for 1 min on ice. Cells were then washed with cold PBS, filtered using a 40 µm cell strainer and centrifuged at 650 x g for 8 min. FACS buffer containing PBS supplemented with 0.5% BSA was used to resuspend the cells. Prior to sorting, cells were stained with Calcein Violet 50 AM (65‐0854‐39, Thermo Fisher Scientific). After sorting on a SH800S sorter (Sony Biotechnology, 100 µm sorting chip), the number of viable cell count was obtained using a Neubauer hemocytometer and Trypan Blue staining. Cells were processed according to the manufacturer's Chromium Next GEM Single cell 3′ v3.1 protocol using the 10X Genomics platform. Sequencing of the libraries was performed on an Illumina NovaSeq 6000 instrument at FGCZ using the 10X Genomics specifications for paired‐end reads: R1 = 28, i7 = 8, i5 = 0, R2 = 90. For each sample, an average read depth of ≈50 000 per cell was achieved.

### scRNA‐Seq Analysis of In Vitro Samples

The CellRanger pipeline v7.0.0^[^ ^82^ ^]^ was utilized to demultiplex samples, align reads to the bovine reference genome assembly Bos_taurus_UMD_3.1 (Ensembl release 92), process cell barcodes, and count unique molecular identifiers (UMIs). Filtered feature‐barcode count matrices were subsequently processed and analyzed using the R package Seurat v4.2.1.^[^ ^83^, ^84^ ^]^ In addition, the R package scDblFinder^[^ ^85^ ^]^ was employed to detect and remove doublets from each sample. For quality control, cells were excluded from downstream analyses if they exhibited unique feature counts below 1000 or above 6000 (or 7500 for myoblast medium), UMI counts exceeding 30,000 (40,000 for iMPC medium or 60,000 for myoblast medium), or mitochondrial gene counts over 5%. Filtered cells were log normalized per condition and integrated using the canonical correlation analysis method implemented in Seurat.^[^ ^83^, ^84^ ^]^ The integrated cells were subsequently clustered using the Louvain algorithm at a resolution of 0.5 based on the top 30 principal components. Cluster marker genes were identified using differential expression analysis via Wilcoxon rank‐sum test (criteria: |avg_log2FC| > 0.25 and adjusted p‐value < 0.01). Based on established marker genes from the literature,^[^ ^30^, ^86^, ^87^, ^88^ ^]^ cell clusters were annotated into broad cell types. Differential expression analysis (Wilcoxon rank‐sum test; |avg_log2FC| > 0.25 and adjusted p‐value < 0.05) was conducted for each cell type to compare the different conditions. Integrated dSMCs from the iFR^hi^, iFRC, and Conv. Diff. conditions were re‐clustered using the same pipeline. Data visualization was carried out with the R package SCpubr v2.0.^[^ ^89^ ^]^ Raw gene counts were aggregated across cells, either at the dSMC sub‐type or medium sample level to generate pseudo‐bulk data.

### RNA velocity analysis using scRNA‐Seq

The raw reads were aligned to the Bos taurus reference genome (build UMD_v3.1) and gene‐level expression was quantified using STARSolo (Version 2.7.8a),^[^ ^90^ ^]^ employing gene models from Ensembl release 92 in CB_UMI_Simple mode with multi‐gene UMI filtering. Spliced and unspliced read quantification was carried out by setting the soloFeatures flag to “Gene Velocyto.” Following alignment, cell barcodes were filtered to retain only those used in the analyses before. In R (version 4.3.0), gene‐level, spliced, and unspliced count matrices were merged into a single SingleCellExperiment object (version 1.22.0).^[^ ^33^ ^]^ Log‐normalization of the gene‐level counts was performed using the scuttle R package (version 1.10.1),^[^ ^91^ ^]^ and the top 2000 highly variable genes were identified with scran (version 1.28.1),^[^ ^92^ ^]^ followed by dimensionality reduction using scater (version 1.28.0).^[^ ^91^ ^]^ Finally, RNA velocity calculations were carried out in dynamical mode using Velociraptor (version 1.10.0) as a wrapper for the Python package scvelo (version 0.2.2),^[^ ^93^ ^]^ employing the top 1000 highly variable genes and 50 nearest neighbors.

### scRNA‐Seq Analysis of In Vivo‐Derived Muscle Samples

For in vivo skeletal muscle tissue analysis, MA and MM skeletal muscle samples were obtained from the slaughterhouse and processed on the same day. For analysis of scRNA‐Seq, CellRanger pipeline v7.1.0^[^ ^82^ ^]^ was used for sample demultiplexing, read alignment against the bovine reference genome assembly Bos_taurus_UMD_3.1 (Ensembl release 92), cell barcode processing and UMI counting. The R package Seurat v4.4.0^[^ ^83^, ^84^ ^]^ was used for processing and analyzing the filtered feature‐barcode count matrices. The R package scDblFinder^[^ ^85^ ^]^ was used to identify and remove doublets from each sample. Cells with unique feature counts below 1000 or above 5000, UMI counts over 20 000, and mitochondrial gene counts greater than 5% were filtered out from the downstream analysis for quality control. Filtered cells were normalized via SCtransform and integrated together via canonical correlation analysis. Normalized cells from integrated samples were clustered together applying Louvain algorithm based on top 30 principal components. To identify marker genes for each cluster, differential gene expression analysis (Wilcoxon rank‐sum test; |avg_log2FC| > 0.25 and adjusted p‐value < 0.01) was performed. Based on known marker genes from the literature,^[^ ^30^, ^86^, ^87^, ^88^ ^]^ the cell clusters were annotated into broad cell populations. The integrated myogenic cells were re‐clustered using the same pipeline described above. Raw gene counts of skeletal muscle cells were aggregated together to generate pseudo bulk data. Pearson correlation coefficient was computed between pseudo bulk in vivo skeletal muscle cells and in vitro dSMC sub‐types or media‐specific dSMCs based on log normalized overlapping gene expression. Data normality was assessed using the Kolmogorov‐Smirnov test.

### Fabrication of Molds and Scaffolds for 3D Printing

Molds and scaffolds were designed using the CAD software (Fusion360) and exported as STL files. The constructs were printed by Spectroplast using their Silicone 3D Printing Service (Spectroplast, 8952 Schlieren, Switzerland). TrueSil silicone materials with a Shore hardness of 20A were selected for molds, and 35A for scaffolds. Prior to use, all constructs were thoroughly washed with PBS and sterilized by autoclaving at 121 °C for 15 min.

### Generation of 3D Tissue‐Engineered Muscle Rings

Printed molds were incubated with an anti‐adherence rinsing solution (07010, STEMCELL Technologies) for 15 min and then washed once with PBS. Muscle rings were generated by adapting previously published methods for engineered 3D skeletal muscles.^[^ ^94^, ^95^ ^]^ Expanded myogenic cells were dissociated in 0.25% Trypsin‐EDTA to create a single‐cell suspension, then ≈6 × 10^6^ cells were resuspended in 79.2 µL of low glucose DMEM (31 885 023, Thermo Fisher Scientific), mixed with 150 µL of fibrinogen (8 mg mL^−1^) (F8630‐1G, Sigma–Aldrich), 0.8 µL of thrombin (0.5 U per mg of fibrinogen) (T4648‐1KU, Sigma–Aldrich), and 120 µL of 30% (v/v) Matrigel (FAL354234, Corning) to form a gel‐cell solution at a concentration of 15 × 10^6^ cells per mL. A volume of 150 µL of the gel‐cell solution was injected into each mold and incubated at 37 °C for 1 h in 12‐well plates. Following incubation, 2 mL of growth medium containing 1.5 mg mL^−1^ 6‐aminocaproic acid (ACA) was added to the injected molds. The following day, muscle rings were carefully transferred from the molds to the scaffolds for structural support. After 2 days of cell growth, the proliferation medium was replaced with 2 mL of differentiation medium supplemented with ACA (2 mg mL^−1^) for 5 days, with daily medium changes. Images of the entire muscle rings were taken on a Nikon SMZ‐1270 stereomicroscope with a Qimaging Retiga R1 camera.

### Contraction Displacement Analysis

Contraction videos of muscle rings were recorded using Eclipse Ti2‐E Inverted Microscope (Nikon) at 10–25 frames per second (fps) to ensure adequate temporal resolution of contractile events. Videos were saved in an “mp4” format for subsequent analysis using PIVlab (https://ch.mathworks.com/matlabcentral/fileexchange/27659-pivlab-particle-image-velocimetry-piv-tool-with-gui). The contraction videos were converted into sequential image frames using the built‐in functions of the software. Each frame was subdivided into interrogation windows to calculate displacement and velocity vectors. The PIV settings were configured to use a two‐phase interrogation process, with interrogation window sizes set to 64 pixels in the first phase and 32 pixels in the second. The local displacement of interrogation windows between consecutive frames was determined using the Fast Fourier Transform (FFT) algorithm to perform cross‐correlation and identify the maximally correlated window pairs. The computed motion vector fields provided quantitative maps of contractile movement, displaying displacement at each time point. Post‐processing steps were performed to calibrate scale and improve data quality, including noise reduction using smoothing filters and the exclusion of low‐contrast and excessively bright areas. Data was exported as “.mat” files. These “.mat” files were then used to extract the mean velocity magnitude over time using custom MATLAB code. The extracted data was then saved into Excel sheets and plotted to visualize the velocity magnitude (µm/s) over time.

### Statistical Statement

Statistical analyses were performed to compare myotube surface area and RT‐qPCR gene expression values between the small molecule containing media (iFR^hi^ or iFRC) and the conventional differentiation medium. Standard deviation was used for these comparisons. The number of samples used is indicated in the respective figure legend. One‐way ANOVA followed by post‐hoc Dunnett's test was applied for multiple comparisons. Myotube surface area was analyzed using GraphPad Prism version 10 and RT‐qPCR data were analyzed using the R packages stats and multcomp. All statistical analyses were visualized using GraphPad Prism version 10. For omics datasets, statistical analysis was carried out as described in the respective Method sections, or figure legends.

## Conflict of Interest

O.B.‐N. is an inventor on a pending patent related to the production of iMPCs filed by the General Hospital Corporation (Pub. No. US20230042917A1, Appl. No. US17/846,556, filed June 22, 2022). The remaining authors declare no competing interests.

## Author Contributions

C.L.T. and A.G. contributed equally to this work. The study was conceptualized by CLT, AG, and OBN. Experiments involving isolation and differentiation of myoblasts, as well as characterization through microscopy, RT‐qPCR or immunostaining were performed by CLT. Additionally, multiomics analyses involving bulk RNA‐Seq and proteomics were performed by CLT and AG. Multiomics analyses involving scRNA‐Seq were performed by CLT, AG and FN. Finally, experiments involving tissue engineering of 3D bovine muscle rings were performed by CLT and AKK. Contraction displacement analysis was done by AKK. The manuscript was written by CLT, AG and OBN. The study was supervised by OBN.

## Supporting information



Supporting Information

Supplemental Movie 1

Supplemental Movie 2

Supplemental Movie 3

Supplemental Movie 4

Supplemental Movie 5

Supplemental Movie 6

Supporting Information

## Data Availability

The Bulk RNA‐Seq and scRNA‐Seq datasets can be accessed in the Gene Expression Omnibus (GEO) repository under accession number GSE262758. The mass spectrometry proteomics (LC‐MS) dataset has been deposited to the ProteomeXchange Consortium via the PRIDE^[^ ^69^ ^]^ partner repository and given the dataset identifier PXD051019. Published in vivo bovine muscle datasets were downloaded from GEO using the accession numbers GSE211428^[^ ^40^ ^]^ and GSE205347,^[^ ^41^ ^]^ respectively.
