# Supplementary material for: Enhanced Media Optimize Bovine Myogenesis in 2D and 3D Models for Cultivated Meat Applications
Source: Adv Sci (Weinh). 2025 Jul 28;12(35):e13998. doi: 10.1002/advs.202413998 (PMC12462954; doi:10.1002/advs.202413998)
Supplement: Supplementary file 1 — Supporting Information [file ADVS-12-e13998-s007.pdf]

ADVANCED SCIENCE
Open Access

Supporting Information

for *Adv. Sci.*, DOI 10.1002/advs.202413998

Enhanced Media Optimize Bovine Myogenesis in 2D and 3D Models for Cultivated Meat Applications

*Christine L. Trautmann, Adhideb Ghosh, Ali Kerem Kalkan, Falko Noé and Ori Bar-Nur**

Supporting Information for

Enhanced Media Optimize Bovine Myogenesis in 2D and 3D Models for Cultivated Meat Applications

Christine L. Trautmann[1*], Adhideb Ghosh[1,2*], Ali Kerem Kalkan[1], Falko Noé[1,2] & Ori Bar-Nur[1#]

[1] Laboratory of Regenerative and Muscle Biology, Institute of Human Movement Sciences and Sport, Department of Health Sciences and Technology, ETH Zurich, Schwerzenbach, 8603, Switzerland

[2] Functional Genomics Center Zurich, ETH Zurich and University of Zurich, Zurich, 8057, Switzerland

*Equal contribution

#Correspondence: Ori.bar-nur@hest.ethz.ch

This file includes:

Supplementary Figures 1- 7

Supplementary Tables 1, 2

Supplementary Movie Legends 1- 6

Supplementary Data Legend 1

Supplementary Figure 1

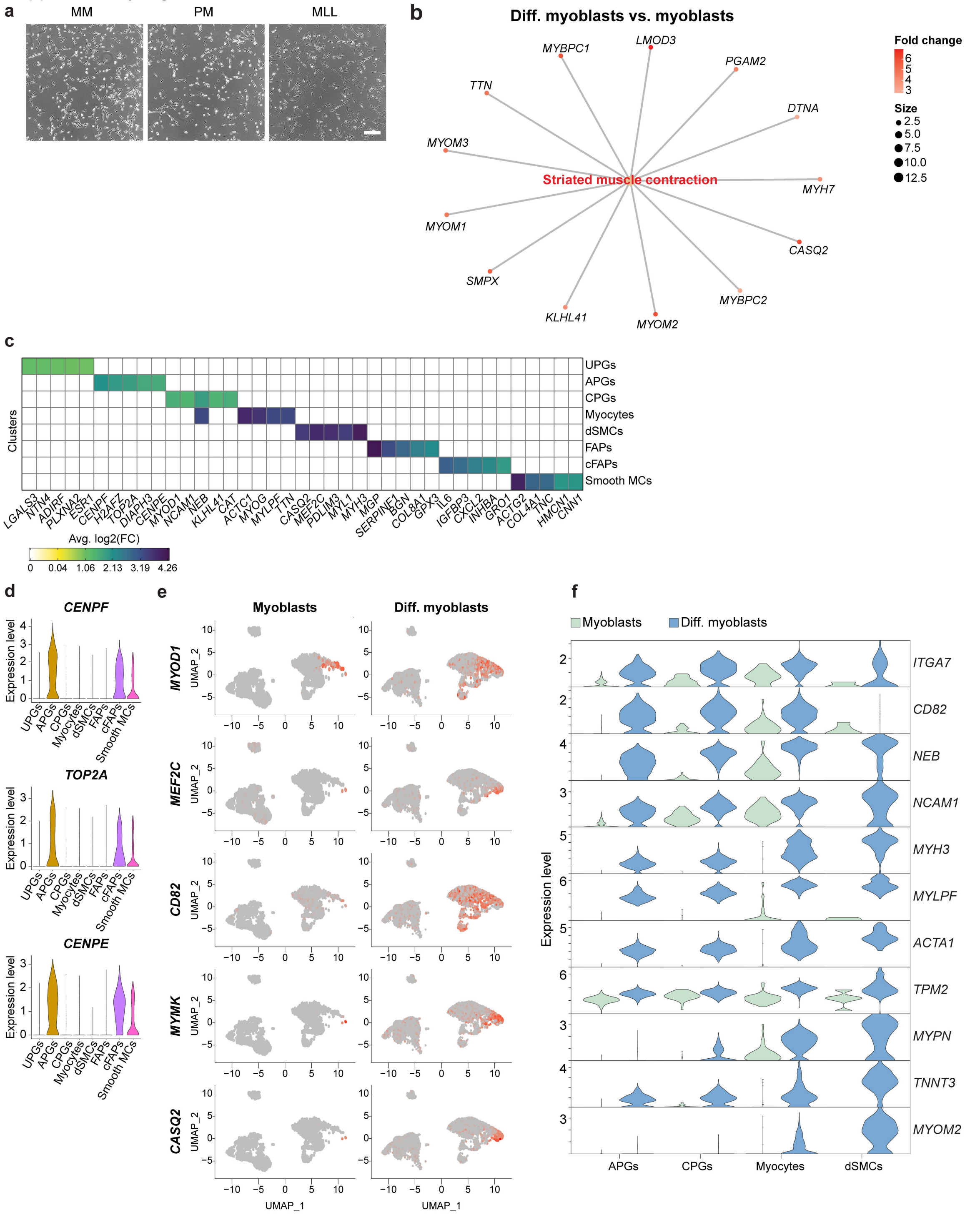

Figure S1: Molecular characterization of conventional bovine myoblast differentiation **(a)** Representative phase contrast images of bovine myoblast lines isolated from the indicated muscle types. The myoblasts are at passage 2, shown one day after cell splitting. Look-up tables (LUTs) were equally adjusted. Scale bar, 250 µm. **(b)** Network plot based on RNA-Seq showing upregulated genes in differentiated myoblasts vs. myoblasts that comprise the GO term pathway "*Striated muscle contraction*". Color scale corresponds to the log-fold change values from the RNA-Seq data. N=3 myoblast lines per group, derived from three different muscle types. The differentiated myoblasts were analyzed at day 15. **(c)** Heatmap based on scRNA-Seq showing the top five marker genes for each one of the 8 specified cell populations.

An integrated dataset of “myoblasts” and “differentiated myoblasts” was used for this analysis. Color scale corresponds to the average log-fold change values. The differentiated myoblast culture, derived using the Conv.Diff. condition, was analyzed at day 10. An MA myoblast line was used for the scRNA-Seq analysis. **(d)** Violin plots showing the expression of the indicated cell cycle-related genes across all cell populations. An integrated dataset of “myoblasts” and “differentiated myoblasts” was used for this analysis. **(e)** UMAP projection of the integrated cells colored by myogenic gene expression in the indicated conditions. **(f)** Violin plots based on scRNA-Seq showing the expression of the indicated myogenic genes in myoblasts and differentiated myoblasts (Conv. Diff. condition) for each specified cell population.

Supplementary Figure 2

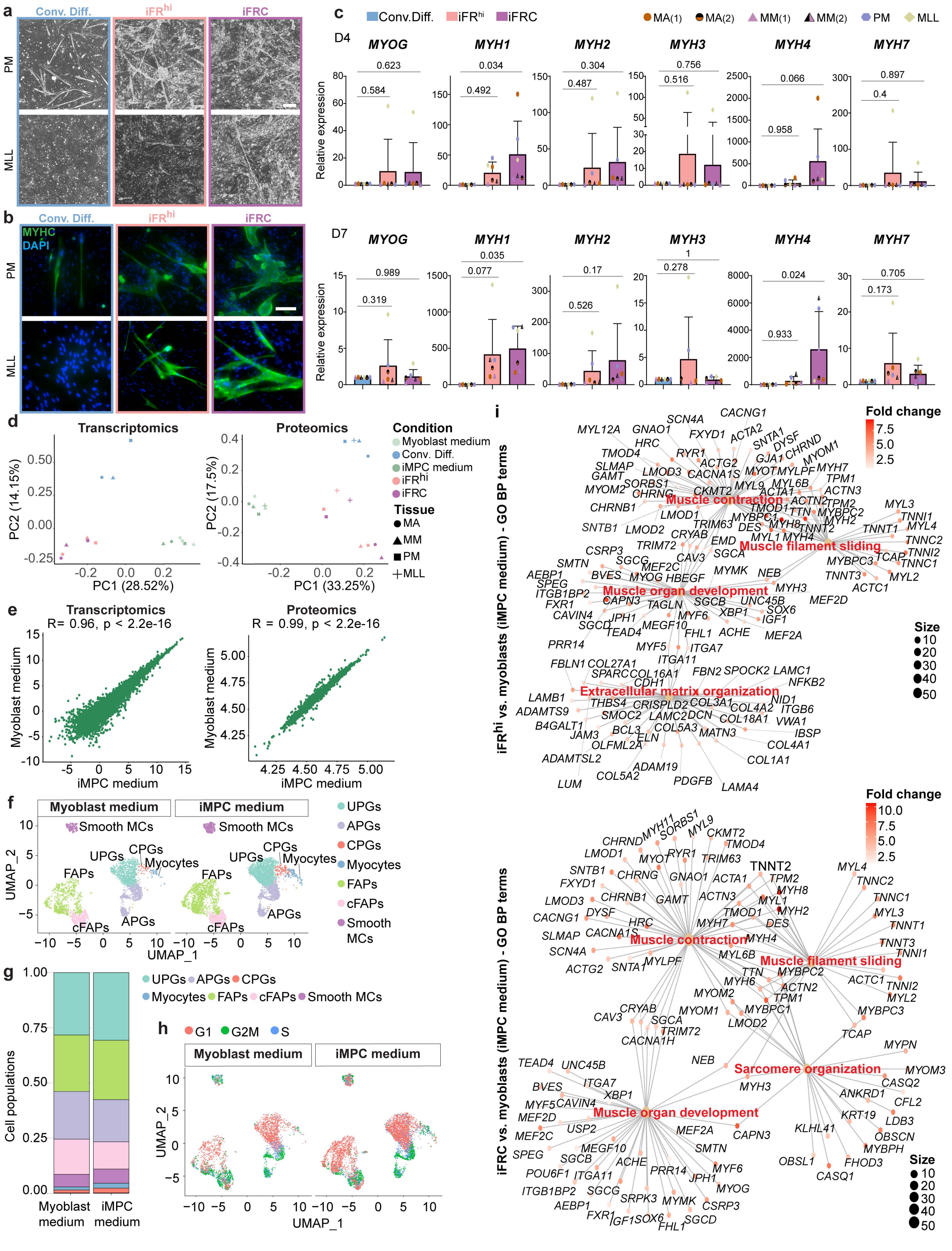

Figure S2: Improved myoblast differentiation with small molecule treatment

(a) Representative phase contrast images of differentiated myoblasts from the indicated muscle types cultured under the specified conditions and imaged at day 10. LUTs were individually adjusted. Scale bar, 250 µm. **(b)** Representative immunofluorescence images for MYHC in the specified myoblast types that have been differentiated using the indicated conditions and stained at day 10. Note that in MLL-derived myoblasts subjected to the Conv.Diff. condition, no MYHC-expressing myotubes were detected in this randomly taken image, in accordance with the low differentiation levels observed with this condition. Nuclei were stained with DAPI. LUTs were individually adjusted. Scale bar, 100 µm. **(c)** qRT-PCR

analysis of the outlined myogenic genes in myoblasts subjected to the specified differentiation conditions and analyzed at the indicated times. N=6 different myoblast lines from four different muscle types as specified. Error bars denote standard deviation (SD). Statistical significance was determined by one-way ANOVA, and the indicated p values were computed using post-hoc Dunnett's test. **(d)** PCA based on bulk RNA-Seq and LC-MS datasets as indicated. Sample dots are colored by different conditions and specified by skeletal muscle type. N=15 for RNA-Seq and N=18 for LC-MS (see Table S2 for specific details). **(e)** Scatter plots based on RNA-Seq (left) and LC-MS (right) datasets showing similar gene and protein expression pattern between the indicated conditions. Pearson correlation coefficient and p values are provided. For RNA-Seq, N=3 myoblast lines for either myoblast medium or iMPC medium. For LC-MS, N=2 for myoblast medium and N=4 for iMPC medium. **(f)** UMAP projection based on scRNA-Seq of integrated cells from the indicated conditions colored by specific cell populations. An MA myoblast line was used for this scRNA-Seq analysis. **(g)** Bar plots based on scRNA-Seq showing relative cell population distribution across the specified conditions. **(h)** UMAP projection of integrated cells colored by cell cycle states in the specified conditions. **(i)** Network plot showing GO biological process (BP) terms for upregulated genes in the iFR^{hi} and iFRC conditions vs. myoblasts cultured in the iMPC medium. Color scale corresponds to the log-fold change values obtained from bulk RNA-Seq data. N=3 myoblast lines per group, derived from three different muscle types and analyzed at day 15.

Supplementary Figure 3

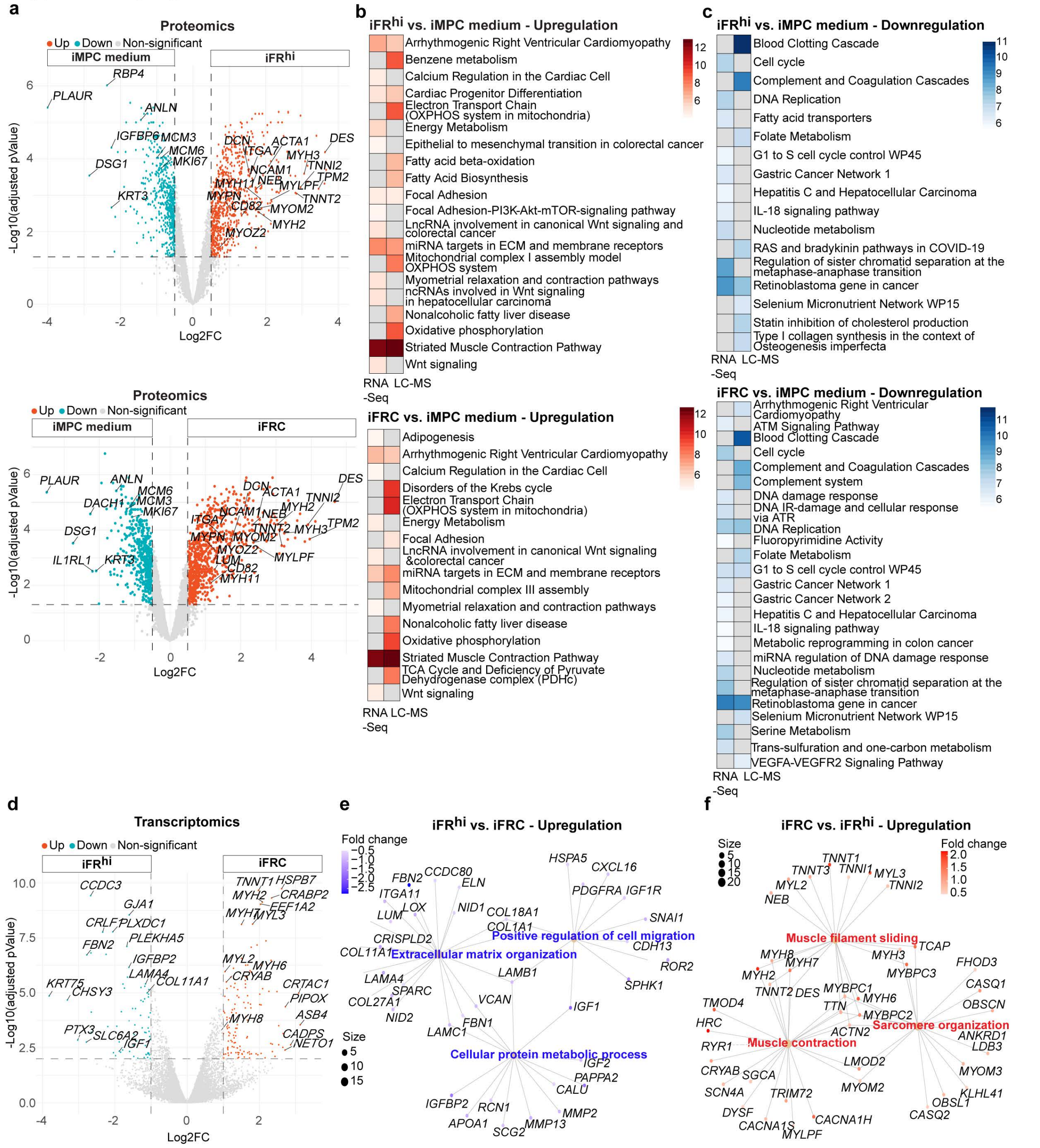

Figure S3: Dissecting the effect of small molecules on myoblast differentiation

(a) Volcano plots based on LC-MS showing DEPs between the indicated conditions. Significant DEPs (|log2FC| > 1, FDR < 0.05) are shown as colored dots. N=4 myoblast lines per group, derived from four different muscle types. **(b)** Heatmaps showing upregulated WikiPathways terms in iFR^{hi} and iFRC conditions vs. iMPC medium, based on significant DEGs and DEPs as indicated. Color scale corresponds to normalized combined score from the pathway enrichment analysis. For RNA-Seq, N=3 myoblast lines per group, derived from three different muscle types. For LC-MS, N=4 myoblast lines per group, derived from four different muscle types. **(c)** Heatmaps showing downregulated WikiPathways terms in iFR^{hi} and iFRC treated

cells vs. iMPC medium based on significant DEGs and DEPs as indicated. Color scale corresponds to normalized combined score from pathway enrichment analysis. For RNA-Seq, N=3 myoblast lines per group, derived from three different muscle types. For LC-MS, N=4 myoblast lines per group, derived from four different muscle types. **(d)** Volcano plot showing DEGs between the indicated conditions. Note several myogenic differentiation genes that are upregulated in the iFRC vs. iFR^{hi} conditions. Significant DEGs ($|log2FC| > 1$, $p < 0.01$) are shown as colored dots. N=3 myoblast lines per group, derived from three different muscle types. **(e)** Network plot showing upregulated genes in the iFR^{hi} vs. iFRC condition for the indicated GO term biological processes. Color scale corresponds to the log-fold change values from the RNA-Seq data. N=3 myoblast lines per group, derived from three different muscle types. **(f)** Network plot showing upregulated genes in the iFRC vs. iFR^{hi} condition for the indicated GO term biological processes. Color scale corresponds to the log-fold change values from the RNA-Seq data. N=3 myoblast lines per group, derived from three different muscle types and analyzed at day 15.

Supplementary Figure 4

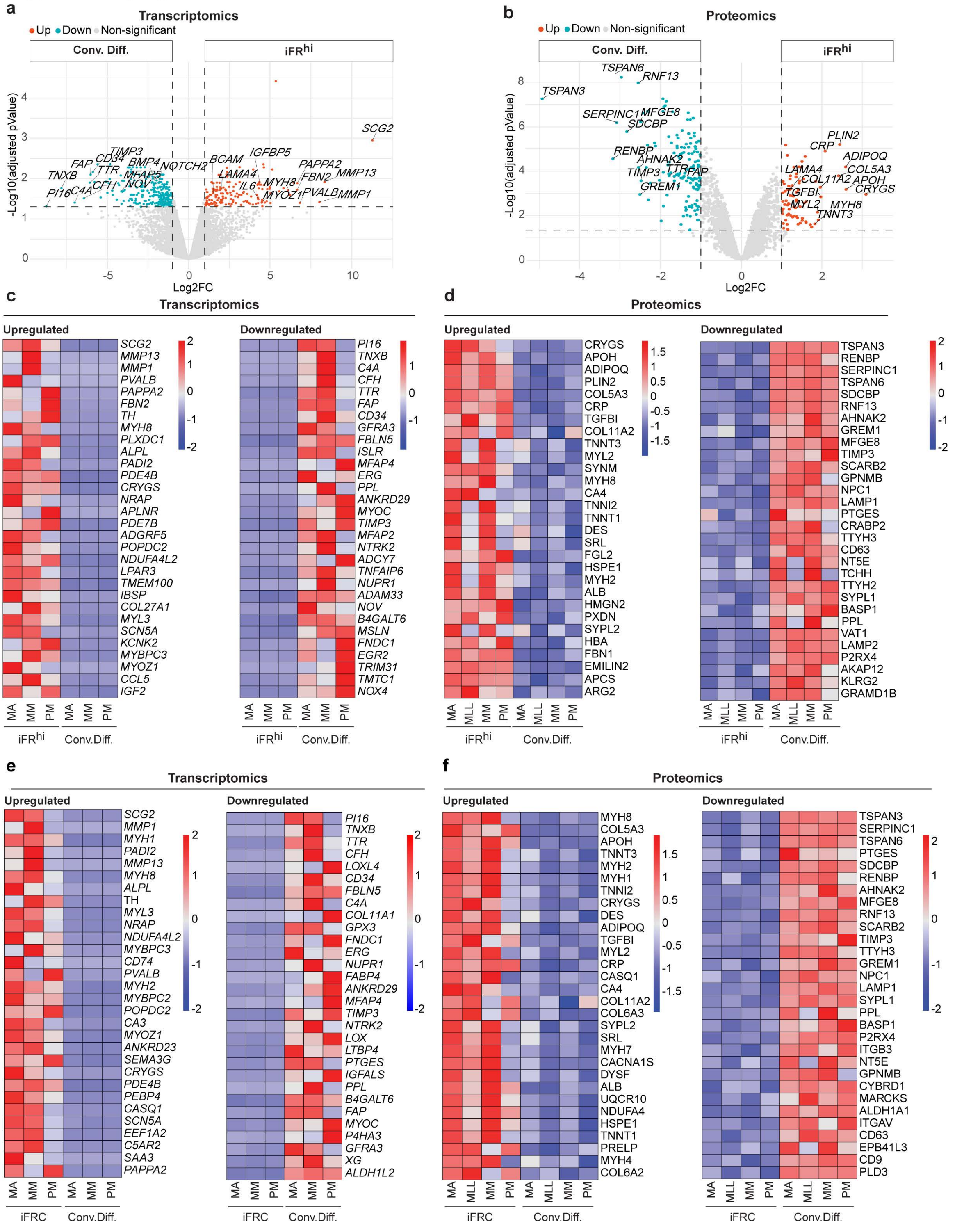

Figure S4: Differentially expressed myogenic markers induced by small molecules
(a) Volcano plot based on RNA-Seq showing DEGs between the indicated conditions. Significant DEGs (|log2FC| > 1, FDR < 0.05) are shown as colored dots. N=3 myoblast lines per group, derived from three different muscle types. **(b)** Volcano plot based on LC-MS showing DEPs between the indicated conditions. Significant DEGs (|log2FC| > 1, FDR < 0.05) are shown as colored dots. N=4 myoblast lines per group, derived from four different muscle types. **(c)** Heatmap based on RNA-Seq showing the top 30 up- and down-regulated DEGs in iFR^{hi} in comparison to the Conv.Diff. condition. Color gradient corresponds to scaled normalized gene expression. N=3 myoblast lines per group, derived from three different

muscle types. **(d)** Heatmap based on LC-MS showing the top 30 up- and down-regulated DEPs in iFR^{hi} in comparison to the Conv.Diff. condition. Color gradient corresponds to scaled normalized protein expression. N=4 myoblast lines per group, derived from four different muscle types. **(e)** Heatmap based on RNA-Seq showing the top 30 up- and down-regulated DEGs in iFRC in comparison to the Conv.Diff. condition. Color gradient corresponds to scaled normalized gene expression. N=3 myoblast lines per group, derived from three different muscle types. **(f)** Heatmap based on LC-MS showing the top 30 up- and down-regulated DEPs in iFRC in comparison to the Conv.Diff. condition. Color gradient corresponds to scaled normalized protein expression. N=4 myoblast lines per group, derived from four different muscle types. For all the conditions shown in this figure, RNA-Seq was analyzed at day 15 and LC-MS at day 10 of differentiation.

Supplementary Figure 5

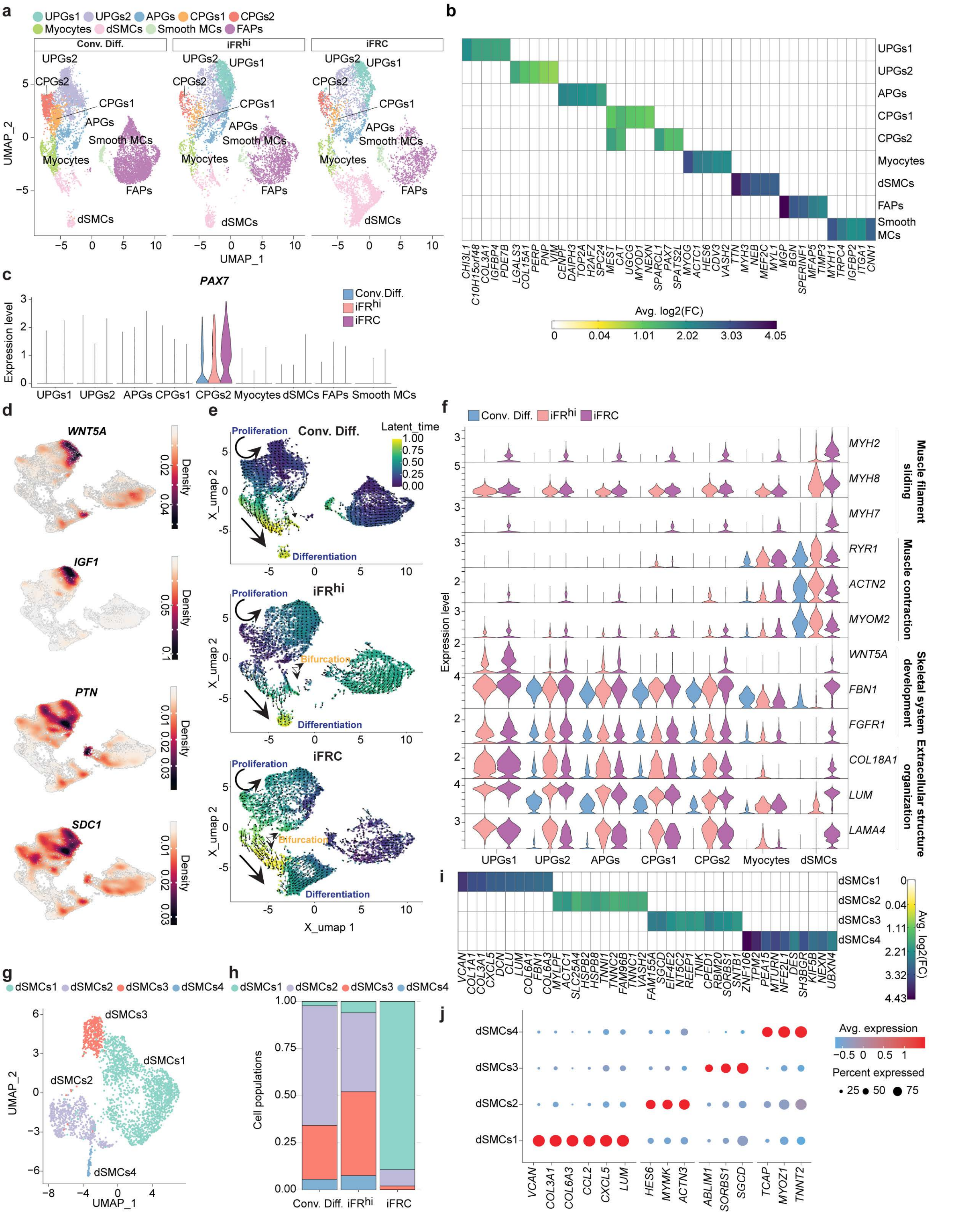

Figure S5: scRNA-Seq analysis of myoblasts subjected to small molecule treatment
(a) UMAP projection based on scRNA-Seq of integrated MA myoblast-derived cells subjected to the indicated differentiation conditions and colored by specific cell populations. Note reduction in FAPs and an increase in dSMCs in the iFRC condition. The cultures were analyzed at day 10. **(b)** Heatmap showing the top five marker genes for each one of the 9 specified cell populations. An integrated dataset of the three differentiation conditions was used for this analysis. Color scale corresponds to the average log-fold change values. **(c)** Violin plot based on scRNA-Seq showing the expression level of *PAX7* across 9 cell populations in MA-derived myoblasts subjected to the indicated conditions. **(d)** UMAP projection of integrated cells colored by the density gradient of the indicated marker genes which are enriched in UPGs1

(top 2) or UPGs1 and UPGs2 (bottom 2). **(e)** UMAP projection of integrated cells for the indicted differentiation conditions colored by latent time as calculated by RNA velocity. **(f)** Violin plots showing expression of DEGs associated with the specified GO terms in the indicated cell populations that are either similar or enriched in the iFR^{hi} and iFRC conditions vs. the Conv. Diff. condition. **(g)** UMAP projection of integrated dSMCs 1-4 colored by specific sub-populations. **(h)** Bar plots based on scRNA-Seq showing relative dSMC sub-population distribution across the specified conditions. **(i)** Integrative heatmap showing the top five marker genes for each dSMC sub-population. Color scale corresponds to the average log-fold change values. **(j)** Dot plot based on scRNA-Seq showing the expression level of representative marker genes for dSMCs1-4.

Supplementary Figure 6

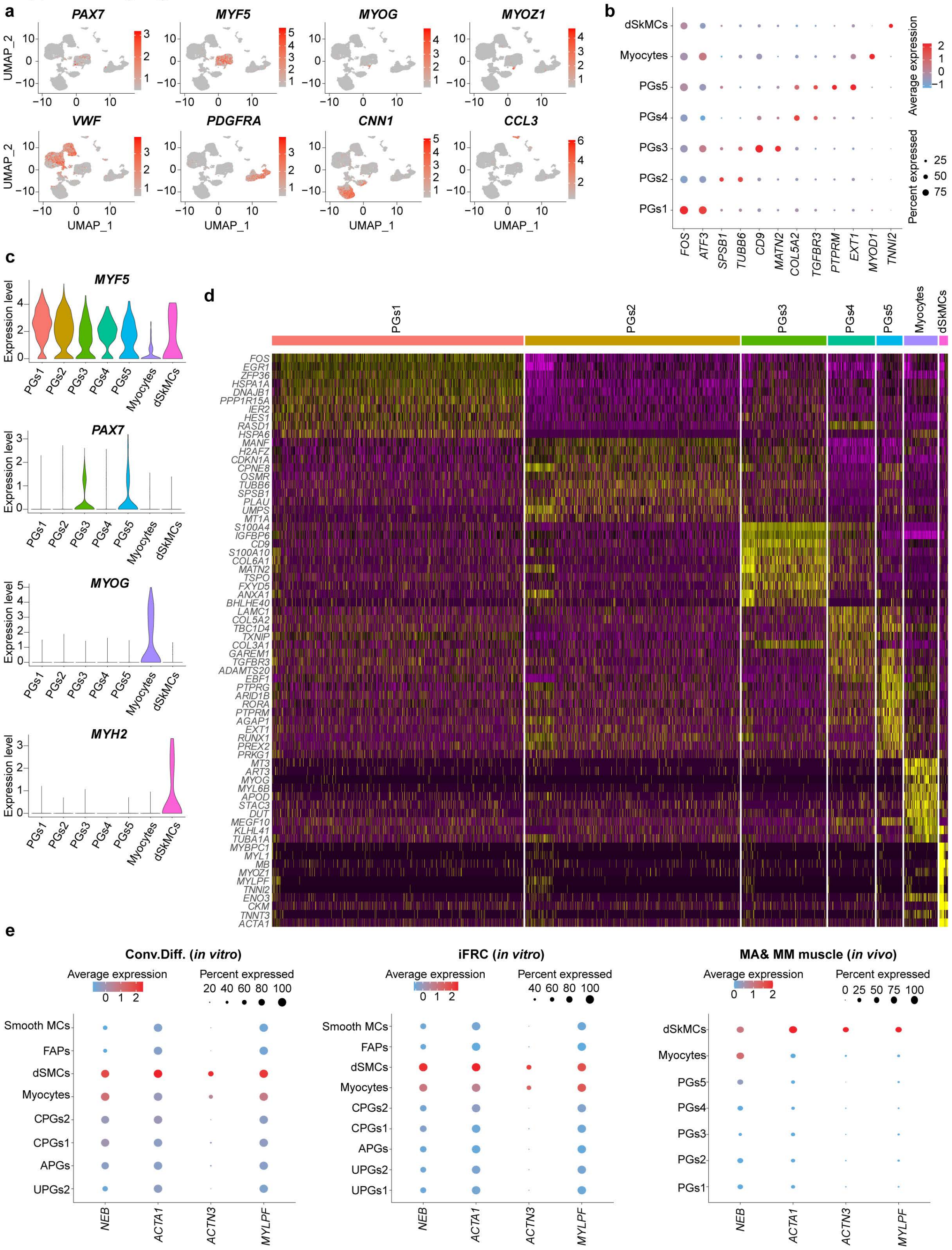

Figure S6: scRNA-Seq analysis of *in vivo*- and *in vitro*-derived muscle cells
(a) UMAP projection of *in vivo*-derived resident muscle cells colored by cell type marker gene expression. The scRNA-Seq data represents the integration of MA and MM muscles. **(b)** Dot plot showing the expression level of marker genes indicative of the annotated myogenic sub-populations. The specified myogenic sub-populations were re-clustered from the total cell populations comprising the integrated MA and MM *in vivo* muscle scRNA-Seq dataset. **(c)** Violin plots exhibiting the expression levels of the indicated genes across the *in vivo*-derived myogenic sub-populations. **(d**) Heatmap showing the expression of the top 10 marker genes indicative of each myogenic sub-population, corresponding to the integrated and re-clustered

MA and MM *in vivo* muscle dataset. **(e)** Dot plots showing the expression levels of the specified skeletal muscle genes for the indicated *in vitro* and *in vivo* samples and conditions.

Supplementary Figure 7

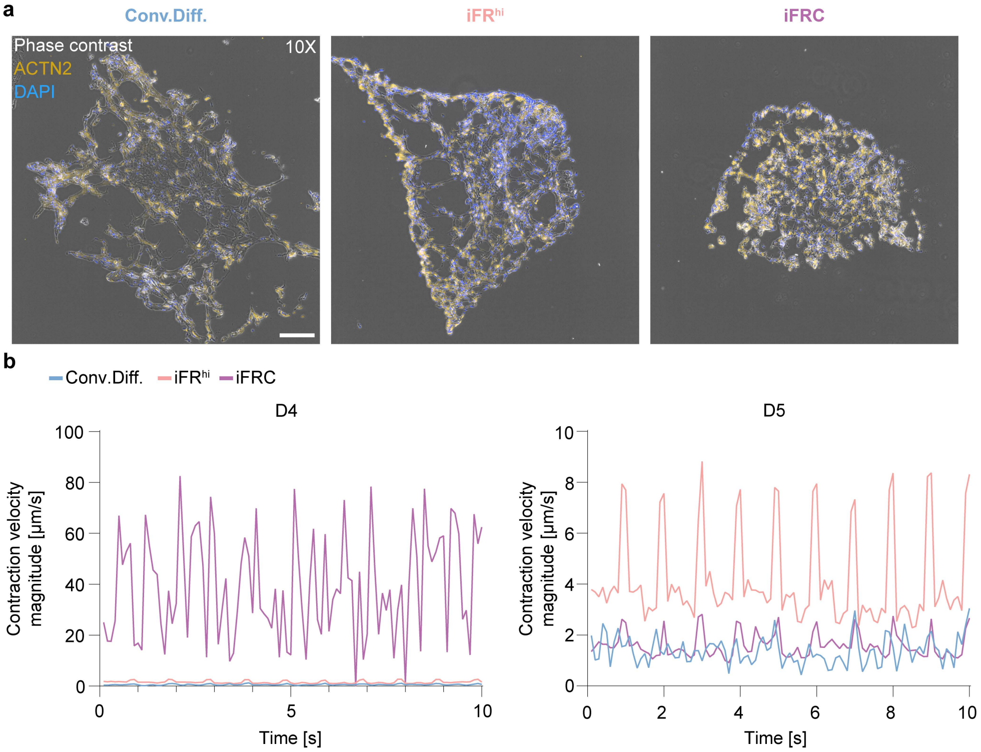

Figure S7: Analysis of 3D tissue-engineered skeletal muscle rings
(a) Phase contrast and immunofluorescence overlay images of muscle ring cross-sections obtained using the indicated differentiation conditions. Note that these images correspond to the images shown in Figure 6c, representing similar images overlayed here with phase contrast images. The muscle rings were immunostained for the myogenic marker ACTN2. LUTs were equal across all conditions. Scale bar, 200µm. **(b)** Graphs showing contraction displacement measurement in MM-2 myoblast-derived muscle rings at the indicated days. Note that myoblasts subjected to the iFRC condition exhibited contractility earlier than the iFR^{hi} condition, and with a higher contraction velocity magnitude at this time point. S, seconds.

Supplementary Tables:

Muscle type	Cut	Origin	Gender	Age (months)	Breed
MA-1	Flank steak	Zurich slaughterhouse	Male	12	Black Angus
MA-2	Flank steak	Zurich slaughterhouse	Female	14	N/A
MA-3	Flank steak	Zurich slaughterhouse	N/A	N/A	N/A
MM-1	Beef cheek	Zurich slaughterhouse	Male	12	Black Angus
MM-2	Beef cheek	Zurich slaughterhouse	Female	14	N/A
MM-3	Beef cheek	Zurich slaughterhouse	Male	12	N/A
PM-1	Filet	Hinwil slaughterhouse	Male	17	Black Angus
MLL-1	Sirloin/Rumpsteak	Hinwil slaughterhouse	Female	27	Brown Limousin

Supplementary Table 1: A summary of the myoblast lines used in this study, including their origin and characteristics. Each myoblast line corresponds to a distinct muscle isolated from a different cow. The muscles were isolated from 6 different cows. The provided information is based on data received from the donating slaughterhouses, when available. MA: *M. abdominis*, MM: *M. masseter*. PM: *M. p. major*: MLL: *M. l. lumborum*. N/A, not available.

Assay / Condition	Myoblast medium	iMPC medium	Conv.Diff condition	iFR^{hi} condition	iFRC condition	N (total)
scRNA-Seq	MA-3	MA-3	MA-3	MA-3	MA-3	5*1 = 5
Bulk RNA-Seq	MA-3<br>MM-3<br>PM-3	MA-3<br>MM-3<br>PM-3	MA-3<br>MM-3<br>PM-3	MA-3<br>MM-3<br>PM-3	MA-3<br>MM-3<br>PM-3	5*3 = 15
Proteomics (LC-MS)	MA-3<br>MM-3	MA-3<br>MM-3<br>PM-1<br>MLL-1	MA-3<br>MM-3<br>PM-1<br>MLL-1	MA-3<br>MM-3<br>PM-1<br>MLL-1	MA-3<br>MM-3<br>PM-1<br>MLL-1	4*4 + 1*2 = 18
Surface area analysis of $MYHC^+$ myotubes	MA-1<br>MA-2<br>MM-1<br>MM-2<br>PM-1<br>MLL-1	MA-1<br>MA-2<br>MM-1<br>MM-2<br>PM-1<br>MLL-1	MA-1<br>MA-2<br>MM-1<br>MM-2<br>PM-1<br>MLL-1	MA-1<br>MA-2<br>MM-1<br>MM-2<br>PM-1<br>MLL-1	MA-1<br>MA-2<br>MM-1<br>MM-2<br>PM-1<br>MLL-1	5*6 = 30
RT-qPCR for myogenic genes at day 4 and 7			MA-1<br>MA-2<br>MM-1<br>MM-2<br>PM-1<br>MLL-1	MA-1<br>MA-2<br>MM-1<br>MM-2<br>PM-1<br>MLL-1	MA-1<br>MA-2<br>MM-1<br>MM-2<br>PM-1<br>MLL-1	3*6 = 18
RT-qPCR for myogenic genes in 3D muscle rings			MM-1<br>MM-2	MM-1<br>MM-2	MM-1<br>MM-2	3*2 = 6

Supplementary Table 2: A summary of the myoblast lines and differentiation conditions used throughout the study, including the molecular assays employed for the specified myoblast lines. Myoblasts were isolated from 6 cows as detailed in Table 1. MA: *M. abdominis*, MM: *M. masseter*. PM: *M. p. major*: MLL: *M. l. lumborum*.

Supplementary Movie Legends:

Movie 1 shows MA myoblast-derived myotubes obtained using the Conv. Diff. condition.
Movie 2 shows contractile MA myoblast-derived myotubes obtained using the iFR^{hi} condition.
Movie 3 shows contractile MA myoblast-derived myotubes obtained using the iFRC condition.
Movie 4 shows an MM myoblast-derived muscle ring generated with Conv. Diff.
Movie 5 shows an MM myoblast-derived contractile muscle ring generated with iFR^{hi}.
Movie 6 shows an MM myoblast-derived contractile muscle ring generated with iFRC.

Supplementary Data Legend:

Data S1 contains cell-type specific top 20 marker genes for integrated scRNA-seq data, related to Figure 1,3,4,5 and Figure S1,2,5,6.
